# Phylogeographic Relationships among *Bombyx mandarina* (Lepidoptera: Bombycidae) Populations and Their Relationships to *B. mori* Inferred from Mitochondrial Genomes

**DOI:** 10.3390/biology11010068

**Published:** 2022-01-03

**Authors:** Min-Jee Kim, Jeong-Sun Park, Hyeongmin Kim, Seong-Ryul Kim, Seong-Wan Kim, Kee-Young Kim, Woori Kwak, Iksoo Kim

**Affiliations:** 1 Experiment and Analysis Division, Honam Regional Office, Animal and Plant Quarantine Agency, Gunsan 54096, Korea; minjeekim3@korea.kr; 2 Department of Applied Biology, College of Agriculture & Life Sciences, Chonnam National University, Gwangju 61186, Korea; jungsun5009@naver.com (J.-S.P.); cjdfydxyz@naver.com (H.K.); 3 Department of Agricultural Biology, National Academy of Agricultural Science, Rural Development Administration, Wanju Gun 55365, Korea; ksr319@korea.kr (S.-R.K.); tarupa@korea.kr (S.-W.K.); applekky@korea.kr (K.-Y.K.); 4 Hoonygen, Seoul 08592, Korea; woori@hoonygen.com

**Keywords:** *Bombyx mori*, *Bombyx mandarina*, domesticated silkworm, wild silkworm, progenitor, phylogeny, whole-genome sequencing

## Abstract

**Simple Summary:**

*Bombyx mandarina* (Lepidoptera: Bombycidae), the presumed ancestor of *B. mori*, has long been a subject of study to illustrate the geographic variation in relationship to origin of *B. mori*. We report 37 mitochondrial genome (mitogenome) sequences of *B. mori* strains obtained by whole-genome sequencing and four of *B. mandarina* individuals obtained by Sanger sequencing from South Korea. These mitogenome sequences were combined with 45 public data to uncover the population genetic and phylogenetic relationships among regional populations of *B. mandarina* and their relationships to *B. mori*. A substantial genetic reduction in *B. mori* strains compared to the *B. mandarina* was detected, with the highest diversity detected in the Chinese origin *B. mori*. Chinese *B. mandarina* were divided into northern and southern groups, largely concordant to the Qinling–Huaihe line, and the northern group was placed as an immediate progenitor of monophyletic *B. mori* strains in phylogenetic analyses. The enigmatic South Korean population of *B. mandarina*, which has often been regarded as a closer genetic group to Japan, was most similar to the northern Chinese group, evidencing substantial gene flow between the two regions. This is the first report that the South Korean *B. mandarina* are closer genetically to the northern Chinese group.

**Abstract:**

We report 37 mitochondrial genome (mitogenome) sequences of *Bombyx mori* strains (Lepidoptera: Bombycidae) and four of *B. mandarina* individuals, each preserved and collected, respectively, in South Korea. These mitogenome sequences combined with 45 public data showed a substantial genetic reduction in *B. mori* strains compared to the presumed ancestor *B. mandarina*, with the highest diversity detected in the Chinese origin *B. mori*. Chinese *B. mandarina* were divided into northern and southern groups, concordant to the Qinling–Huaihe line, and the northern group was placed as an immediate progenitor of monophyletic *B. mori* strains in phylogenetic analyses, as has previously been detected. However, one individual that was in close proximity to the south Qinling–Huaihe line was exceptional, belonging to the northern group. The enigmatic South Korean population of *B. mandarina*, which has often been regarded as a closer genetic group to Japan, was most similar to the northern Chinese group, evidencing substantial gene flow between the two regions. Although a substantial genetic divergence is present between *B. mandarina* in southern China and Japan, a highly supported sister relationship between the two regional populations may suggest the potential origin of Japanese *B. mandarina* from southern China instead of the Korean peninsula.

## 1. Introduction

In addition to its importance in silk production for more than 5000 years, the domesticated silkworm, *Bombyx mori* (Lepidoptera: Bombycidae), is used as an important research and industrial resource in several fields of biology, such as genetics, genomics, and biomedicine, as well as for industrial and food industry applications [1,2,3,4,5,6]. The long history of its importance is reflected in the form of diverse strains preserved in Asia and Europe with a broad array of independent mutations (e.g., 4000 strains) [7].

As in other domesticated animals, *B. mori* also has long been a subject of domestication-related issues [8,9,10,11]. Generally, there is a strong consensus primarily based on archeological data that *B. mori* evolved from the wild silkworm, *Bombyx mandarina*, and its place of origin is in China among its distributional range, which also includes Korea, Japan, and India [12,13]. Indeed, this notion is further supported by earlier studies wherein the chromosome number of *B. mandarina* inhabiting China and Ussuri (northwest of North Korea in China and Russia) is *n* = 28, which is the same as that of *B. mori* [14,15,16,17], whereas that in Japan and South Korea is *n* = 27 [14]. Regarding the difference in chromosome number between the two species, Banno et al. [18] showed the fusion of two chromosomes contained in *B. mori* in the *B. mandarina* with *n* = 27.

In addition to the earlier chromosomal study [14], chromosomal studies using different geographic samples of *B. mandarina* from South Korea [17,19,20] and the pattern of a short retroposon-like insertion in the arylphorin gene [17] have found a closer genetic relationship of *B. mandarina* between South Korea and Japan. On the other hand, phylogenetic analysis using ~1.4 kb of *Mariner*-like elements has shown a closer genetic relationship of *B. mandarina* inhabiting South Korea to China, instead of Japan [21]. Furthermore, the *EcoR*I digesting site, located between the 5.8S and 28S rRNA genes [20] also supports the findings of Kawanishi et al. [21]. However, these findings are inconsistent with their chromosomal analysis, which showed *n* = 27 in the *B. mandarina* inhabiting South Korea [20]. With regards to this contradicting result, on one hand, an independent evolutionary event between the chromosome fusion and rDNA-type separation has been suggested [20]. On the other hand, the distribution of both chromosomal types in South Korea based on unpublished data (*n* = 27 and 28, including hybrid-type individuals) has also been suggested [20,21]. Thus, controversy remains with regards to the genetic status of the wild silkworm inhabiting South Korea.

Mitochondrial genomes (mitogenomes) and segments of mitochondrial DNA (mtDNA) alone or in combination with nuclear genes have also been employed to understand genetic relationships among geographic samples of *B. mandarina* and to trace ancestral populations of *B. mori* strains [3,10,22,23]. These studies consistently and strongly support a closer genetic relationship of *B. mandarina* inhabiting China instead of Japan to *B. mori* [10,22], and this result is also supported in earlier studies using segments of mtDNA [8,9]. Recently, an extensive phylogenetic analysis using 55 mitogenome sequences composed of 39 *B. mori* strains from diverse phenotypes and origins, including one South Korean strain and 16 *B. mandarina* individuals collected from China and Japan, along with two from northern China, was reported [23]. The study evidenced that *B. mandarina* distributed in northern China is an immediate progenitor of *B. mori*, separated from those inhabiting southern China and Japan [23]. However, no previous mitogenome- and segments of mtDNA-based studies have included any *B. mandarina* individuals from South Korea, assuming genetic similarity of *B. mandarina* in South Korea to that in Japan [10,22,23], possibly due to earlier studies, which revealed an identical chromosome number of *B. mandarina* between the two countries [14,17,19,20].

**Figure 1:**
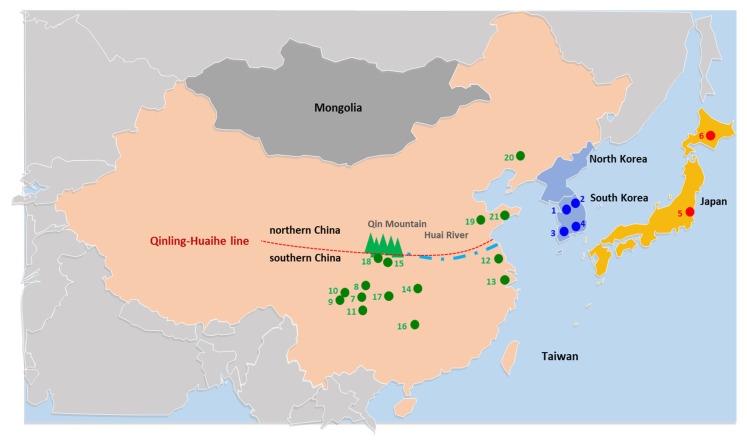
Sampling locations of *Bombyx mandarina* sequenced in this study and obtained from public data. General locality names are as follows: 1, Gapyeong; 2, Inje; 3, Gwangju; and 4, Sacheon (from South Korea); 5, Tsukuba and 6, Hokkaido (from Japan); 7, Ziyang; 8, Nanchong; 9, Hongya; 10, Pengshan; 11, Luzhou; 12, Yancheng; 13, Suzhou; 14, Yichang; 15, Ankang; 16, Hunan; 17, Chongqing; 18, Shiquan (from southern China); and 19, Qingzhou, Shandong; 20, Shenyang, Liaoning; and 21, Haiyang (from northern China). Coordinates of the sampling sites for South Korean individuals are as follows: 1, Gapyeong, 37°57′54.5″ N, 127°26′35.7″ E; 2, Inje, 37°55′46.1″ N, 128°23′15.9″ E; 3, Gwangju, 35°10′47.4″ N, 126°56′27.6″ E; and 4, Sacheon, 35°04′22.6″ N, 128°00′37.9″ E.

In this study, we report 37 mitogenome sequences of *B. mori* strains preserved in Korea, which are composed of a diverse phenotype, including trimolters and tetramolters. We also report four mitogenome sequences of *B. mandarina* collected from different regions in South Korea (two from both southern and northern regions; Figure 1). These 41 sequences were combined with 45 publicly available sequences, which consisted of 28 strains of *B. mori* preserved mainly in China as well as in other countries, including two from South Korea and 17 *B. mandarina* individuals collected from different regions in China and two from Japan. We analyzed the mitogenomic characteristics, population genetic diversity, population genetic structure, and phylogeny to uncover relationships among regional populations of *B. mandarina* and their relationships to *B. mori*.

## 2. Materials and Methods

### 2.1. Sampling and Sequencing

Both adults and larvae of *B. mandarina* were collected either by light trap or hand from mulberry leaves, respectively, during September 2014 and October 2020 from the different localities (Inje, Gapyeong, Sacheon, and Gwangju), which are 100.9 km (Inje vs. Gapyeong) to 421.23 km (Inje vs. Gwangju) apart in South Korea (Figure 1). The specimens were deposited at the Chonnam National University under the voucher numbers CNU12048, CNU12051, CNU12047, and CNU14308 (Table S1).

To sequence the mitogenome of *B. mandarina* by Sanger’s method, genomic DNA was extracted either from two hind legs of adults or from the thorax and abdomen of larvae after the midgut and head were removed using a Wizard^TM^ Genomic DNA Purification Kit (Promega, Madison, WI, USA) according to the manufacturer’s instructions. Three primer sets that amplified three long overlapping fragments (LFs; LF1, LF2, and LF3) were adapted from Kim et al. [24]; LF1, LF2, and LF3 amplify *COI* to *ND4* (~7.2 kb), *ND5* to *lrRNA* (~6.7 kb), and *lrRNA* to *COI* (~4.8 kb), respectively. Amplification of the LFs was conducted using LA Taq^TM^ (Takara Biomedical, Tokyo, Japan) under the following conditions: 96 °C for 2 min, 30 cycles of 98 °C for 10 s and 48 °C for 15 min, and a final extension step of 72 °C for 10 min. Thereafter, these amplicons were used as templates to amplify 26 overlapping short fragments (SFs) using AccuPower^®^ PCR PreMix (Bioneer, Daejeon, Korea) under the following conditions: initial denaturation for 5 min at 94 °C, followed by 35 cycles of 30 s at 94 °C, 1 min at 48–52 °C, 1 min at 72 °C, and a final 7 min extension at 72 °C. The 26 primer set that amplified each of the 26 SFs was also adapted from Kim et al. [24]. Five SFs (SF10, SF11, SF13, SF14, and SF26) were sequenced after cloning due to dubiety in direct sequences, whereas the remaining SFs were sequenced directly after PCR purification. Cloning was carried out using a pGEM-T Easy vector (Promega, Madison, WI, USA) and HIT DH5α competent cells (Real Biotech Co., Banqiao City, Taiwan). The resultant plasmid DNA was isolated using the Plasmid Mini Extraction Kit (Bioneer, Daejeon, Korea). DNA sequencing was conducted using the ABI PRISM^®^ BigDye^®^ Terminator v3.1 Cycle Sequencing Kit and the ABI PRISM^TM^ 3100 Genetic Analyzer (PE Applied Biosystems, Foster City, CA, USA). All the products were sequenced from both directions.

### 2.2. Mitochondrial Genome Assembly and Annotation

Recently, we conducted whole genome sequencing for the Korean endemic strain, Goryeosammyeon, which molts three times, lasting to the fourth instar, along with 36 *B. mori* strains preserved in Korea (4 trimolters and 32 tetramolters) using Illumina NextSeq (13 strains), MGISeq 2000 (23 strains), and PacBio Sequel (the strain Goryeosammyeon) sequencing systems. Each mitogenome was assembled from these genomic data, and raw data availability can be found in Table S1.

For mitogenome assembly using short read data from both NextSeq and MGISeq platforms, the quality of the raw sequencing data (reads) was assessed using the FastQC [25]. Sequencing artifacts and low-quality bases were trimmed off from the raw reads by Skewer [26]. To improve the assembly contiguity, the sequencing error correction process was performed using Karect [27]. Reconstructions of mitogenomes were conducted using MITObim [28]. For MITObim assembly, seed sequences were isolated from the read mapping results to the known mitogenome of *B. mori* (unpublished, GenBank acc. no. AF149768) using BWA-MEM [29].

For mitogenome assembly using the long reads that were generated by the PacBio Sequel sequencing system for the *B. mori* strain Goryeosammyeon, mitochondrial reads were isolated prior to assembly from whole genome data by mapping to the known *B. mori* reference mitogenome (unpublished, GenBank acc. no. AF149768) using BLASR (matched length > 500 bp) [30]. Reconstruction of the mitogenome was performed using the CANU [31] with the default option. After assembly, raw reads were mapped to the constructed mitogenome using BLASR, and assembly polishing was conducted using Arrow (https://github.com/PacificBiosciences/pbbioconda, accessed on 19 Febraury 2021), which is a specifically designed polishing tool for Pacbio read. To improve the assembly quality of the constructed mitogenomes, mapped reads were manually checked for each constructed mitogenome using IGV [32].

For the four *B. mandarina* sequences, which were obtained by Sanger’s method, individual SF sequences were assembled manually into the complete mitogenome using SeqMan in the DNASTAR package (DNASTAR, Madison, WI, USA) by following the protocols presented by Cameron [33]. To assess the completeness of the assembly, an annotation process was carried out for constructed mitogenomes using the MITOS2 web server ([34]; http://mitos2.bioinf.uni-leipzig.de/index.py, accessed on 19 Febraury 2021). The 41 mitogenomes constructed in this study have been deposited in GenBank (Table S1).

### 2.3. Collection of Public Data

For the comparative analysis, publicly available mitogenome sequences of the *B. mori* strains and *B. mandarina* individuals were downloaded from the GenBank database (Table S1). These consisted of 28 domesticated silkworm strains that originated from eight countries (China, Cambodia, India, Italy, Korea, Japan, Laos, and Russia) and Europe [10,35,36,37,38,39,40,41,42] and 17 wild silkworms collected from China and Japan [10,23,43,44,45,46] (Table S1). Consequently, 45 public data entries and our own 41 mitogenome sequences, totaling 86 mitogenome sequences of *Bombyx*, were used for the genomic, phylogeographic, and phylogenetic analyses, along with two *Bombyx* species downloaded as outgroups in phylogenetic analyses [47,48] (Table S1). Sampling localities of *B. mandarina* in previous and current studies are provided in Figure 1.

For further comprehensive comparison of the A + T-rich region structure, publicly available sequences of *B. mandarina* were downloaded from GenBank (Table S2). These included 5 individuals from Japan and 12 from China, respectively, which were originally reported by Sun et al. [22].

### 2.4. Sequence Alignments

To generate a dataset to perform population genetic and phylogenetic analyses, whole mitogenome sequences were aligned with the G-INS-i method using MAFFT ver. 7 [49]. Ambiguous regions, such as microsatellite-like AT-repeat regions located between *ND3* and *trnA*, *trnH,* and *ND4*, and within the A + T-rich region were additionally aligned manually by sight. Consequently, 16,466 bp, including gaps, were generated. We adopted this alignment method to avoid conflict among annotations performed by different authors and to save most variable sites located in tRNA, intergenic spacer sequences (ISSs), and the A + T-rich region, which could be informative for population genetic analyses. Alternatively, as a method of typical alignment, nucleotide sequences of 13 protein-coding genes (PCGs) were individually aligned based on codon positions using RevTrans ver. 2.0 [50]. To decide the codon position, the nucleotide sequences of each PCG were translated based on the genetic code for invertebrate mtDNA. Similarly, 2 rRNA and 22 tRNA genes were also individually aligned with the Q-INS-i method using MAFFT ver. 7 [49]. The A + T-rich region was aligned using the default method using MAFFT ver. 7 [49] and then corrected by sight. Subsequently, individual genes and the A + T-rich region were concatenated into a total alignment (15,940 bp, including gaps) using SequenceMatrix ver. 1.8 [51]. From the two alignment sets, we obtained consistent results in the majority of analyses, but haplotype numbers for population analyses were reduced in the latter method mainly due to the exclusion of substantial lengths of ISSs. Thus, we only provided the results obtained from the former method. Sequence alignment has been published on Mendeley Data (doi: 10.17632/rcp3xf2s56.1). The A/T contents of the whole mitogenome, each gene, and the A + T-rich region were calculated using EditSeq in the DNASTAR packages (Madison, WI, USA) [52]. Gene overlap and intergenic-spacer sequences were counted manually.

### 2.5. Genetic Diversity

For population genetic analysis, all 86 mitogenome sequences were compared to select haplotypes using DnaSP [53], but all mitogenome sequences differ from each other, presenting 86 haplotypes. Genetic diversity estimates were obtained by grouping each *B. mori* and *B. mandarina* population on the basis of their country of origin. *B. mandarina* individuals from China were further divided into southern and northern groups based on a previous phylogenetic study, in which *B. mandarina* collected in China showed two subtypes according to the Qinling–Huaihe line, which divides eastern China into northern and southern regions [23]. This grouping scheme was further applied to other population analyses. Haplotype diversity (*H*) and nucleotide diversity (π) were obtained using Arlequin ver. 3.5 [54] according to the method proposed by Nei [55]. Necessary files are provided in Dataset 1. Maximum sequence divergence within given groups was estimated via extracting the within-group estimates of unrooted pairwise distances using PAUP ver. 4.0 b [56].

### 2.6. Population Structure and Gene Flow

In order to uncover the genetic structure existing in *B. mori* strains and *B. mandarina* individuals, Bayesian Analysis of Population Structure (BAPS) ver. 6.0 [57] was used, with a linked locus option and a codon model. In one analysis, each *Bombyx* species was treated as a separate group, and in another analysis only *B. mori* strains were included, grouping them based on their origin. The analyses were performed with *K* values ranging from 1 to 20, with 10 replicate runs for each *K* value, and optimal clusters were identified based on the maximum log marginal likelihood values.

Population migration rate and genetic distance were estimated for *B. mandarina* populations on the basis of country of origin from subroutines in Arlequin ver. 3.5 [54]. Pairwise genetic distance (*F*_ST_) and a permutation test of the significant differentiation of the pairs of populations (1000 bootstraps) were obtained following the approach described in Excoffier et al. [58] and the distances between DNA sequences were calculated by the Kimura 2-parameters method [59]. Pairwise *F*_ST_ values were used to estimate per generation migration rate, *Nm* (the product of the effective population size *Ne* and migration rate, *m*) based upon the equilibrium relationship: *F*_ST_ =1/(2*Nm* + 1). The degree of *Nm* was visualized as a heatmap using Python 3.5.2, seaborn 0.7.1, numpy 1.12.0, and pandas 0.19.2 (Python Software Foundation, Beaverton, OR, USA).

To detect and plot the relationships among *B. mori* and *B. mandarina* groups, principal coordinate analysis (PCoA) [60] was performed via covariance, with standardization of the genetic distances (*F*_ST_) using GenAlEx ver. 6.5 with default parameters [61]. To increase the robustness of the analysis, each population with more than two haplotypes was included in the PCoA. Therefore, *B. mori* strains originating from Cambodia, Laos, and India were excluded and one *B. mori* strain from Italy was grouped with those from Europe. 

Unrooted pairwise genetic distance among *B. mori* strains and *B. mandarina* individuals was calculated using PAUP ver. 4.01b10 [56]. The degree of individual differentiation was visualized as a heatmap using the online tool Heatmapper ([62]; http://www.heatmapper.ca, accessed on 1 April 2021).

### 2.7. Phylogenetic Relationships

To infer phylogenetic relationships among 86 mitogenome sequences of the two species with two outgroup species (*B. huttoni* and *B. lemeepauli*; Table S1) the maximum likelihood (ML) and Bayesian inference (BI) methods were performed using MrBayes ver. 3.2.7 [63] and RAxML ver. 8.2.10 [64], respectively, which are implemented in CIPRES Portal ver. 3.1 [65]. For BI analysis, two independent runs of four incrementally heated Markov and Monte Carlo chains (one cold chain and three hot chains) were simultaneously run for one million generations, with tree sampling conducted every 1000 generations. The first 25% of the sampled trees was discarded as burn-in with 48 h of maximum running time. The average of the split frequencies under 0.01 was the criterion to decide the reach of convergence from two simultaneous runs. For ML analysis, the RAxML algorithm was applied, which uses a “rapid” bootstrapping approach and searches for the best-scoring tree. Confidence values for BI trees were obtained from the Bayesian posterior probabilities (BPPs), and those for ML trees were determined with 1000 bootstrap (BS) iterations. Substitution model selection, which was conducted by the comparison of AIC scores [66] using Modeltest ver. 3.7 [67], provided the GTR substitution model with Gamma-distributed rate heterogeneity (G) and invariable sites (I) (GTR + G + I) and was applied to both ML and BI analyses.

### 2.8. Bayesian Skyline Plot Analysis

In order to assess past timing and magnitude in female effective population size (*Ne*) of *B. mandarina*, we used the Bayesian skyline plot (BSP) method [68] implemented in BEAST2 [69]. First, divergence time estimation was conducted using a strict global clock assumption in BEAST2, in order to obtain a more reasonable sense of the variability in divergence times for *B. mandarina* lineages. GTR + I + G with four gamma rate categories was used as a substitution model and the prior gammaShape, growthRate, and proportionInvariant.s were set to exponential, respectively. The necessary file is provided in Dataset 2. The property of convergence of the MCMC analysis was decided after inspection of the run in Tracer 1.7.2 (http://tree.bio.ed.ac.uk/software/tracer/, accessed on 3 December 2021). The first 10% of the BEAST2 MCMC run was discarded as burn-in, and the remaining samples were summarized using TreeAnnotator in BEAST2. The resulting consensus tree was checked using FigTree ver. 1.4.4 (http://tree.bio.ed.ac.uk/software/figtree/, accessed on 4 December 2021). Second, the changes in effective population size were investigated to reconstruct the demographic dynamics. For this GTR nucleotide substitution model, a strict clock [70], a substitution rate of 7.563 × 10^−7^ (estimated in the divergence time analysis), and coalescent Bayesian skyline with random tree were selected. The same parameters used in divergence time estimation were used to run the MCMC for BSP analysis. BSP outputs were visualized with Tracer ver. 1.7.2.

## 3. Results

### 3.1. Mitochondrial Genome Composition and Organization

The 37 mitogenomes of *B. mori* strains obtained in this study ranged in size from 15,640 (JAM143) to 15,689 bp (JAM126), with the A/T content ranging from 81.33% (JAM143) to 81.41% (JAM126). Four mitogenomes of *B. mandarina* sequenced in this study ranged in size from 15,657 (collected in Inje) to 15,710 bp (collected in Sacheon) with the A/T content ranging from 81.38% (collected in Gapyeong) to 81.44% (collected in Sacheon) (Table S3). These 41 mitogenomes have typical gene sets (two rRNAs, 22 tRNAs, and 13 PCGs) and a major non-coding A + T-rich region. The codon number was identical at 3733 in *B. mori* strains and *B. mandarina* individuals analyzed in the present study. The length of *srRNA* was identical at 781 bp in all sequenced *B. mori* strains, but was 780 or 781 in *B. mandarina*. The size of *lrRNA* differed slightly among strains and individuals (1376–1379 bp). The length of the A + T-rich region ranged from 489 (JAM320) to 503 bp (JAM315) in *B. mori* strains and 485 to 493 bp in the *B. mandarina*, with the A/T content ranging from 95.33% to 95.63% in *B. mori* and 95.33% to 95.52% in *B. mandarina*, respectively. The longest A + T-rich region detected in *B. mori* JAM315 was mainly due to the presence of extra A/T repeat sequences at the end of the region, closer to *trnM* (data not shown).

In *B. mori* mitogenomes obtained from public data, the majority of strains revealed a highly conserved genome length with an average of 15,661 bp, but Baiyun and Yu5 strains originating from China were slightly shorter at 15,629 and 15,644 bp, respectively (Table S3). On the other hand, *B. mandarina* from public data showed somewhat higher length variation than our *B. mandarina* sequences (15,657–15,710 bp), ranging in size from 15,662 to 15,928 bp in whole genomes. The largest size was detected in two *B. mandarina* individuals originating from Japan (both 15,928 bp), and this has stemmed mainly from the unusually longer A + T-rich region at 747 bp in an average of 512 bp among *B. mandarina* individuals sequenced in this study as well as those from public data. The number of codons in the *B. mori* strains differed between studies. Our annotation resulted in all *B. mori* strains having 3733 codons, whereas those from public data were annotated as having 3720 or 3714 codons. The difference mainly comes from the designation of different positions as potential start and stop codons between studies. Start codons for *ND2*, *COIII*, *ND3*, *ND4*, and *ND5* were 1–7 codons post-positioned, whereas the stop codon for *ND4* was 4 or 2 codons post-positioned compared to our annotation in the genomes (data not shown).

**Figure 2:**
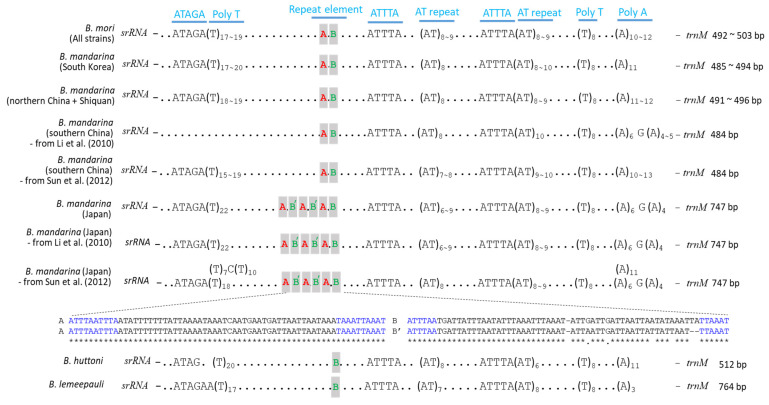
Schematic illustration of the A + T-rich region of *Bombyx mori* and *B. mandarina*. The presented nucleotides indicate conserved sequences, such as the ATAGA sequences and abutting poly-T stretches, ATTTA sequences, AT repeats, ATTTA and abutting AT repeats, poly-T stretches, and poly-A stretches. Dots between sequences indicate omitted sequences. Subscripts indicate the repeat number. The 126 bp repeat element, comprised of subunit A (~64 bp) and subunit B (~62 bp), each of which is flanked by 10 bp perfect inverted repeats or 6 bp perfect inverted repeats (blue color), is presented. Note that all *B. mandarina* from Japan have a triplicated 126 bp repeat, whereas the remaining *B. mandarina* and *B. mori* have a single 126 bp repeat, and *B. huttoni* and *B. lemeepauli* have only subunit B. Where necessary, the origin of sequences and corresponding references are provided.

The A + T-rich region of both *B. mori* and *B. mandarina* from the current study and public data had several conserved sequence elements, which are interspersed in the regions, such as the ATAGA sequences and abutting poly-T stretches, ATTTA sequences, microsatellite-like AT repeats, ATTTA and abutting TA repeats, poly-T stretches, and poly-A stretches (Figure 2). Overall, these elements were well-conserved in both *B. mori* and *B. mandarina*, with some minor variation. The only exception was 15 mitogenomes of *B. mandarina* from southern China, in which the ATAGA sequences and adjoining poly-T stretches, which are located at the beginning of the region closer to *srRNA*, were lacking [10]. The largest size of the A + T-rich region of *B. mandarina* originating from Japan stemmed from the presence of the tandemly triplicated ~126 bp repeat element comprised of ~64 and ~62 bp subunits (subunits A and B, respectively; Figure 2). Each subunit of the element consisted of the core sequence (~44 bp in subunit A and ~50 bp in subunit B, respectively) flanked by 10 or 6 bp perfect inverted repeats in subunit A or B, respectively. On the other hand, the remaining *B. mandarina* and all *B. mori* strains possessed a single copy of the element.

The gene arrangements of *B. mori* and *B. mandarina* in the present study (Table S4) and their publicly available data (data not shown) were identical to that of ditrysian Lepidoptera that have the order *trnM*-*trnI*-*trnQ* (where the underlining indicates a gene inversion) between the A + T-rich region and *ND2* [71,72], unlike the ancestral arrangement in insect mitogenomes, which have *trnI*-*trnQ*-*trnM* between the regions [73,74]. Eleven PCGs had an identical start codon to their isotype in all *B. mori* and *B. mandarina* analyzed in the current study, whereas one *B. mori* strain (Seon7ho) had ATC, instead of ATG in *CytB* (Table S4). All *B. mori* and *B. mandarina* analyzed in the present study had an atypical CGA start codon for the *COI* gene (Table S4), as they are frequently found in the start region of the lepidopteran *COI* gene [24], but are infrequently designated as alternative start codons other than CGA in the public data of *B. mandarina* (data not shown). TAA functions as a stop codon in 11 genes, but *COI* and *COII* harbored an incomplete stop codon, consisting of a single thymine unanimously in all *B. mori* and *B. mandarina* analyzed in the present study.

### 3.2. Genetic Diversity

Genetic diversity estimation in both *B. mori* and *B. mandarina* showed that *B. mandarina* had substantially higher nucleotide diversity (π) (~9.76-fold higher) than *B. mori* (Table 1). Similarly, the number of polymorphic sites and mean number of pairwise differences were also substantially higher in *B. mandarina* (1493 and 253.3, respectively) than those of *B. mori* strains (615 and 25.43, respectively), suggesting an obvious genetic reduction during the long-held domestication process in *B. mori* strains. In regional samples of *B. mori* strains, π was numerically higher in the order of China (0.002668), South Korea (0.001056), Europe (0.000810), and Japan (0.000756), providing at least twofold larger π in China than any other country strains. This estimate could be limiting and biased, considering that the sample size from other countries, except for South Korea, is small, but it is noteworthy in that the sample size of South Korea is more than twofold larger than that of China. The mean number of pairwise difference also showed the same order. Among the regional populations of *B. mandarina*, those from China also showed numerically the highest π compared to other country populations (0.011620 vs. 0.001923 in South Korea and 0.002896 in Japan). When Chinese samples were divided into a northern and southern group, π was numerically similar between southern China (0.004800) and northern China (0.004706), although the sample size between the two regions may have biased the estimates (11 vs. 4 individuals).

### 3.3. Population Structure and Gene Flow

**Figure 3:**
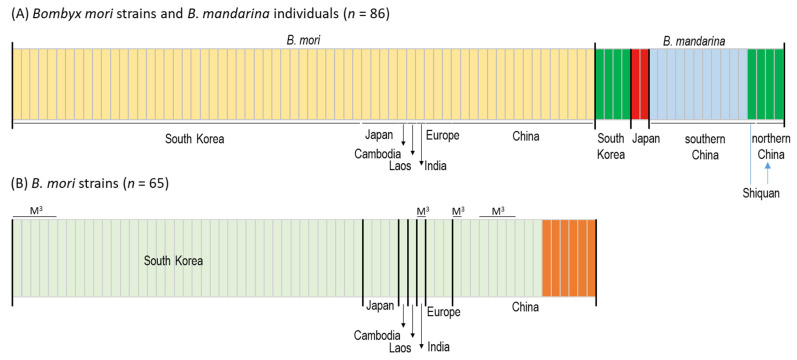
Bayesian clustering analysis. (**A**) Analysis including both *Bombyx mori* strains and *B. mandarina* individuals under the optimum number of clusters (*K*) of 4. (**B**) Analysis including only *B. mori* strains under the optimum number of clusters (*K*) of 2. *B. mori* strains marked with M^3^ are trimolters, whereas the remaining strains are tetramolters. Each vertical bar represents an individual and its associated probability of belonging to the assigned cluster.

An examination of the likelihood scores from 10 replicate runs across *K* values from 1 to 10 for BAPs analysis revealed that available *B. mori* strains and *B. mandarina* individuals were composed of four optimal haplotype clusters (*K* = 4; hereafter referred to as haplogroups). The assignment results of *K* = 4 showed that *B. mori* strains from all countries are composed of a single haplogroup, whereas *B. mandarina* populations are composed of three haplogroups, which are strictly concordant to their geographic origins, except for one individual of *B. mandarina* collected in Shiquan, southern China [43] (Figure 1 and Figure 3). *B. mandarina* individuals from northern China (including Shiquan) and South Korea shared an identical haplogroup (green), whereas individuals from southern China (excluding Shiquan, light blue) and Japan (red) had an independent haplogroup (Figure 3A), suggesting genetic similarity of *B. mandarina* only between northern China (including Shiquan) and South Korea. When *B. mori* strains alone were independently examined for likelihood scores, designating each country strain as a group, two optimal haplogroups were suggested (*K* = 2; Figure 3B). The assignment results of *K* = 2 showed that Chinese *B. mori* strains were divided into two haplogroups (light green and orange), whereas the remaining country strains, including those of South Korea, were assigned to a single haplogroup commonly (light green). Although we included five strains of the tri-molting strains of South Korea and a few of other countries, such as China and India, along with typical tetra-molting strains, any distinction between them was not detected, reinforcing an irrelevant role of mitogenome sequences for moltinism (Figure 3B).

The *F*_ST_ (maximum = 1) between pairs of *B. mandarina* populations ranged from 0.13089 (between South Korea and northern China (including Shiquan); *p* ≤ 0.05)) to 0.93118 (between South Korea and Japan; *p* = 0.069), with a range of *N_m_* from 3.32003 (between South Korea and northern China) to 0.03695 (between South Korea and Japan) (Figure S1). Although the degree of statistical support for *F*_ST_ was not linearized to *N_m_*, possibly due to small sample size (e.g., Hale et al. [75]), particularly in Japan (*n* = 2) the *N_m_* between South Korea and northern China was the highest, providing at least a 22.7-fold higher estimate than any other comparisons, with the 89.85-fold higher estimate than that between South Korea and Japan indicating a higher gene flow between South Korea and northern China (Figure S1).

**Figure 4:**
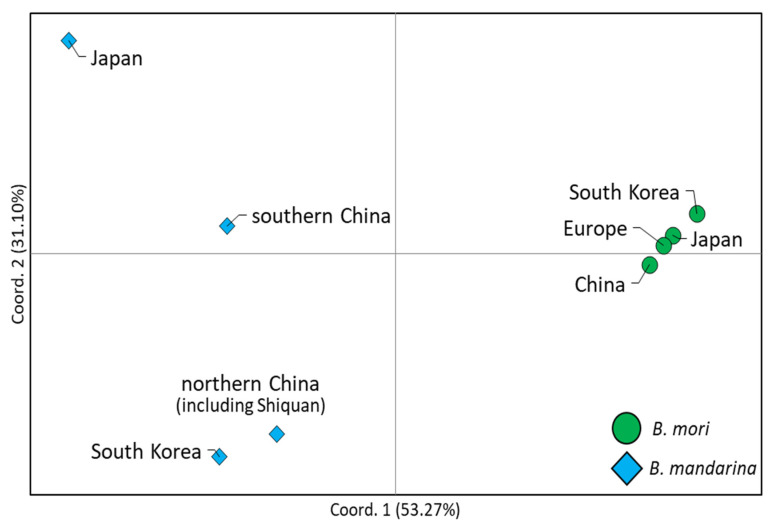
Principal coordinate analysis based on populations. The percentages of variation explained by the first and second components are indicated in the x and y axes, respectively.

A PCoA to scrutinize population relationships showed that *B. mori* strains from different origins formed a closer cluster by both components, which accounted for 53.27% of variation in the first component and 31.1% in the second component, indicating relatively closer genetic relationships among worldwide *B. mori* strains (Figure 4). In the case of *B. mandarina*, those from northern China (including Shiquan) and South Korea formed a closer cluster based on both components, reinforcing a closer genetic relationship of the *B. mandarina* distributed in northern China and South Korea, as has been found in BAPs and *Nm* (Figure 3A and Figure S1). On the other hand, those from southern China and Japan each formed an independent cluster, largely based on the second component, with the placement of the Japan cluster farthest from the remaining *B. mandarina* groups (Figure 4). When *B. mandarina* from Shiquan was included in the southern China group based on the BAPs, no detectable difference in grouping pattern was found, although allocation of the percentage of variation at each component changed slightly (50.74% and 28.87% of variation in the first and second components, respectively; data not shown).

### 3.4. Genetic Distance

**Figure 5:**
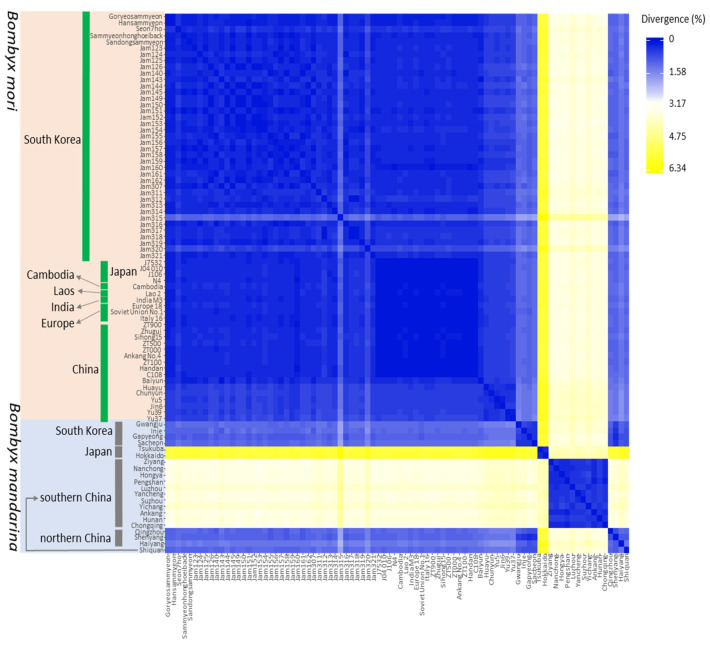
Heatmap showing the unrooted pairwise genetic distance between pairs of haplotypes of *Bombyx mori* and *B. mandarina*. Note that *B. mandarina* collected in Shiquan, which is located in southern China, had higher genetic closeness to those of northern China. Estimates of sequence divergence (minimum, average, and maximum) between all *B. mori* strains and each *B. mandarina* population and between pairs of *B. mandarina* populations are provided in Figure S1.

The heatmap visualizing the genetic differentiation of *B. mori* strains and *B. mandarina* individuals based on unrooted pairwise genetic distance showed that all *B. mori* strains were highly close, forming an immediately closer group with an average distance of 0.383 in the range of 0.006–1.539 (Figure 5 and Figure S2). On the other hand, individuals of *B. mandarina* showed higher genetic variation at 2.631 on average in the range of 0.2–5.89, also indicating a substantial reduction in genetic variation in *B. mori* comparted to *B. mandarina* (Figure 5 and Figure S1B). Regional samples of *B. mandarina* behaved well as different groups, such as South Korean, Japanese, southern Chinese, and northern Chinese groups, but one *B. mandarina* individual collected in Shiquan in southern China showed a higher similarity to the northern Chinese group (Figure 5), as has been shown in BAPs (Figure 3A). Among regional groups, those that originated from South Korea were the closest to *B. mori* at an average distance of 1.174, and northern China (including Shiquan) ranked second at an average distance of 1.274 to *B. mori*, whereas those from southern China and Japan were fairly distant, ranking third (3.402) and last (5.622), respectively (Figure 5 and Figure S2).

Within *B. mandarina*, the genetic distance between regional populations had an average distance of between 0.799 (between South Korea and northern China) and 5.683 (between northern China and Japan), indicating the least divergence between South Korea and northern China (Figure S2B). Comparisons of Japan with other regional samples always showed larger distances than any other comparisons (5.683 to northern China, 4.178 to southern China, and 5.662 to South Korea), with the lowest divergence to southern China, indicating the highest divergence of *B. mandarina* in Japan from others, but those of southern China were closer to Japan than any other regional samples (Figure S2B).

### 3.5. Phylogenetic Relationships

**Figure 6:**
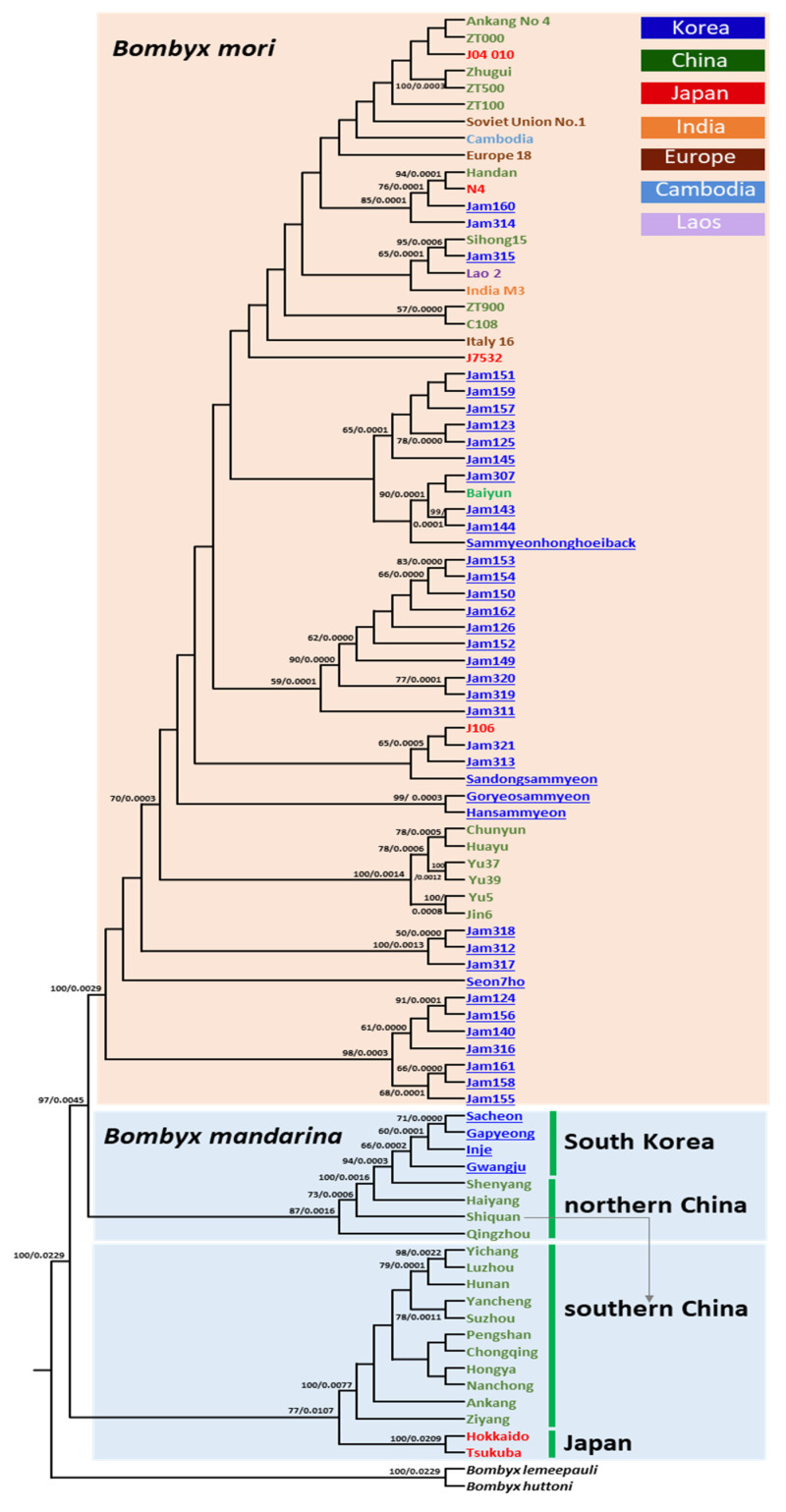
Phylogenetic relationships among *Bombyx mori* and *B. mandarina* haplotypes using the maximum-likelihood method. The numbers at each node specify bootstrap percentages of 1000 pseudoreplicates (**first**) and branch length (**second**). The node supports below 50% were omitted. *B. mori* and *B. mandarina* haplotypes sequenced in this study are underlined. *B. huttoni* and *B. lemeepauli* were used as outgroups.

Phylogenetic analyses using ML and BI methods showed that the *B. mori* strains preserved and originated in different countries consistently formed a strong monophyletic group, separated from *B. mandarina* populations with the highest nodal supports (BS = 100%, Figure 6; BPP = 1.0, Figure S3). Within *B. mori*, however, on the one hand, the analyses revealed consensus topology for a few subgroups of *B. mori* strains, but on the other hand, showed several variable relationships with lower nodal supports for many *B. mori* strains between the two analyses (Figure 6 for ML analysis; Figure S3 for BI analysis). Country-based clustering in *B. mori* strains was detected in a few subgroups, supported with higher nodal supports consistently in both analyses (e.g., BS ≥ 70 by ML and BPP ≥ 0.9 by BI method [76]). For example, JAM124, JAM156, JAM140, JAM316, JAM161, JAM158, and JAM155, which are preserved in South Korea, formed a highly inclusive subgroup, supported strongly in both analyses (BS = 98%, Figure 6; BPP = 1.0, Figure S3). Additionally, several Chinese origin strains, such as Chunyun, Huayu, Yu37, Yu39, Jin6, and Yu5, consistently formed a highly inclusive subgroup (BS = 100%, Figure 6; BPP = 1.0, Figure S3). On the other hand, a few subgroups composed of the *B. mori* strains had different origins. For example, a subgroup consisting of the strains that originated from China (Handan), Japan (N4), and South Korea (JAM160 and JAM314) was strongly supported in both analyses (BS = 85%, Figure 6; BPP = 1.0, Figure S3). Furthermore, JAM315 and Sihong15 originated from South Korea and China, respectively, and formed a subgroup with higher nodal supports at BS = 95% and BPP = 1.0 (Figure 6 and Figure S3). Collectively, *B. mori* strains originating from different countries are phylogenetically mixed within the *B. mori* clade, although several country-based subgroups were detectable, indicating that the evolutionary divergence of each country-preserved *B. mori* strain was not substantial even though they were bred into a diverse strain in each country after domestication.

Within *B. mandarina*, two subgroups were recognized (Figure 6 and Figure S3). All *B. mandarina* haplotypes originating from northern China (Shenyang, Haiyang, and Qingzhou) and South Korea, along with one individual originating from Shiquan in southern China, formed a strong subgroup (BS = 87%; BPP = 1.0). With regards to the relationship with *B. mori*, this subgroup was placed as its sister with the higher nodal supports (BS = 97%; BPP = 1.0) suggesting that an immediate progenitor of *B. mori* could be those distributed mainly in northern China and South Korea. Separated from this subgroup, *B. mandarina* haplotypes that originated from southern China and Japan formed another subgroup with the highest in BI (BPP = 1.0) and higher nodal supports in ML analyses (BS = 77%), respectively, indicating closer phylogenetic relationships of *B. mandarina* individuals that originated from the two regions, although the genetic distance between them was substantial (Figure S2B).

### 3.6. Historical Demography of B. mandarina

**Figure 7:**
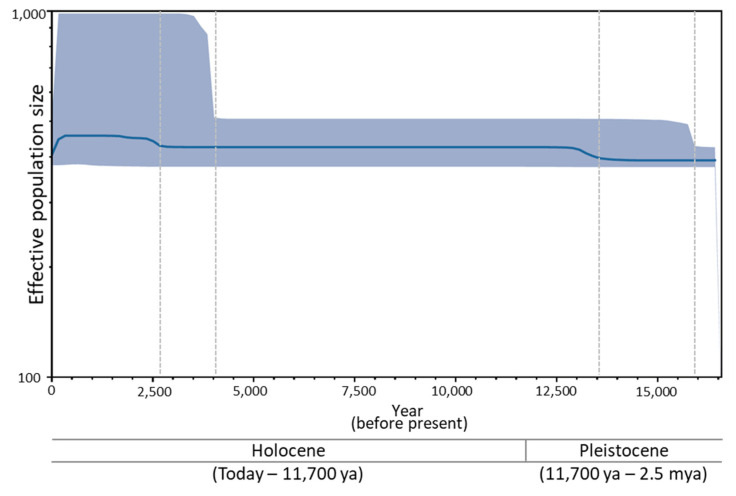
Bayesian skyline plot indicating changes in the effective population size (*Ne*) of *Bombyx mandarina* over time. The shaded area represents the 95% confidence intervals. The vertical dotted lines represent the potential age of the population expansion.

The BSP, which shows the changes in *Ne,* shows relatively constant *Ne* over time in *B. mandarina* populations, but two time periods indicate notable increase (Figure 7). One period ranges approximately from 16,000 to 13,500 in the year before present (YBP) and the other ranges approximately from 4000 to 2800 YBP. A further, obviously larger increase in *Ne* was detected in recent events compared to that of older events.

## 4. Discussion

### 4.1. The A + T-Rich Region

Lepidoptera, particularly Bombycoidea, of which *Bombyx* is a genus, is a well-known insect group that has conserved mitogenomic features, such as gene size, A/T content, usage of start and stop codons, along with conservancy in several sequence elements in the A + T-rich region [23,72,74,77,78,79,80]. In particular, conservancy in a few sequence stretches, and their position related to functional roles, has been well-scrutinized in the A + T-rich region of *B. mori*, as a sole representative of Lepidoptera [81]. These include the ATAGA sequence located immediately at the beginning of the region (closer to *srRNA*), which was suggested to act as a motif sequence that functions as the origin of replication, and the poly-T sequence, located immediately downstream of the motif sequence, which was suggested to function as a structural signal for the recognition of proteins in the initiation of replication for minor-strand mtDNA in *B. mori* [81]. Consequently, these sequences are well-conserved in Lepidoptera, including Bombycoidea, although length variation in the poly-T stretch has been detected (Figure 2) [72,79,80]. Indeed, all *B. mori* and *B. mandarina* sequences analyzed in the present study and the majority of publicly available *B. mori* and *B. mandarina* sequences have these conserved elements. However, 11 *B. mandarina* sequences from southern China were exceptions, lacking both features (Figure 2). We are highly reluctant to speculate that such peculiarity was caused due to alternative elements present in unidentified places in the A + T-rich region, considering a high conservancy of the sequences in Bombycoidea, including other *Bombyx* species [72,79,80]. Furthermore, this feature appears not to be a regional characteristic of *B. mandarina* originating from southern China. Another 12 *B. mandarina* that originated from southern China all had the ATAGA sequence, along with the specified poly-T sequence in conserved locations (Figure 2) [22].

A comparison of all mitogenomes of *B. mori* and *B. mandarina* analyzed in the present study and publicly available mitogenomes of *B. mori* and *B. mandarina* still upheld the uniqueness of the triplicated repeat element only in the Japan-dwelling *B. mandarina*, indicating an evolutionary divergence of the Japanese *B. mandarina* from remaining regions (Figure 2), as has previously been pointed out [8,44]. This result was further supported in the additional sequences of the A + T-rich region from five *B. mandarina* individuals that originated from Japan, although several substitutions and insertions/deletions were present in the repeat sequence elements (Figure 2) [22]. Considering the uniqueness of the repeat element only in the Japanese *B. mandarina*, it was obvious that the Japanese *B. mandarina* acquired the repeat element after splitting from ancestral *B. mandarina*. We further compared this region of other *Bombyx* species, such as *B. huttoni* and *B. lemeepauli*, but these *Bombyx* species only possessed the subunit B as a single copy, lacking subunit A (Figure 2), suggesting that subunit B is the shared ancestral character for the genus *Bombyx*, whereas the subunit A is a derived character that is found only in *B. mori* and its progenitor *B. mandarina*.

### 4.2. Genetic Diversity

Genetic reduction during domestication has been reported in diverse organisms [82,83,84,85,86]. In our mitogenome analysis, loss of 89.75% of nucleotide diversity (π) and 58.81% of polymorphic sites was detected in *B. mori* compared to *B. mandarina* (Table 1). A similar level of reduction also was detected in a previous mitogenome study, where *B. mori* lost 81.61% of π and 61.9% of polymorphic sites compared to *B. mandarina* collected from China [10]. For nuclear DNA, a previous study using nine nuclear gene loci composed of 9268 bp using the coalescent simulation method showed 33–49% reduction in π, which is a similar level to that found in major cultivated crops, and one-half of polymorphic sites in *B. mori* [12]. In the case of gene fragments of *CAD*, 80.56% loss in π was detected in *B. mori* strains preserved in Japan compared to Chinese *B. mandarina* [83]. The extensiveness of genetic reduction in *B. mori* varies among studies, possibly due to differences in the evolutionary nature of employed genes, and the scope of the included taxon, which requires further equivalent comparison. Nevertheless, a typical explanation for a larger reduction in *B. mori* includes artificial selection of the genes related to the domestication process (e.g., *CAD*) and genetic bottleneck, which reduce the genetic diversity presented in the natural populations of ancestral species during the domestication process [12,87,88].

Within *B. mori*, the highest genetic diversity was obvious in the strains preserved in China among country strains, providing more than a twofold larger π and mean number of pairwise genetic distance (Table 1) along with two haplogroups, instead of only one in other country strains, including South Korea (Figure 3B), even though more than twice the number of *B. mori* strains preserved in South Korea compared to China were included in the analysis. These results indicate that the *B. mori* strains from China are genetically diversified, reinforcing the previous finding that the origin of *B. mori* is China [11,22,23]. The insect species introduced to previously unoccupied places often shows lower genetic diversity than that of the original distributional range in both domesticated and natural populations [89,90,91,92]. It is likely that *B. mori* strains domesticated from *B. mandarina* with higher genetic diversity may have allowed the breeding of diversified *B. mori* strains with higher genetic diversity than those in introduced countries, even though introduced countries also bred independent strains. Similarly, other domesticated animals, such as pigs, sheep, goats, cattle, and chickens, have shown that genetic variability declines with increasing distance from the domestication centers [93].

Even though our phylogenetic analysis provided several *B. mori* subgroups, which are supported by higher nodal supports, it is obvious that no *B. mori* subgroups provide clues that indicate a closer affinity to any geographic samples of *B. mandarina* other than the northern Chinese and South Korean group, forming a strong monophyletic group among *B. mori* strains (Figure 6 and Figure S3). A typical interpretation for such a high support for the *B. mori* group could be a monophyletic origin. This implies that data obtained from the present study do not support the multiple origins of *B. mori* in China, which was infrequently suggested by molecular data and archeological records [76]. For this issue, further extensive samplings of *B. mandarina* from a wider region in China and genome-based analysis for the discrimination of subtle changes that occurred in *B. mori* strains must be supplemented for robust inference. Nevertheless, several previous molecular studies supported the monophyletic origin of *B. mori* [23,94].

Among regional populations of *B. mandarina*, the China group also had the highest π, although data from the present study are not unbiased in sample size (two from Japan, four from South Korea, and 15 from China; Table 1). Nevertheless, a supportive result was also reported for *CAD*, which had a further increased sample size, showing approximately sevenfold higher π in Chinese *B. mandarina* compared to that of Japan (0.00725 vs. 0.00112) [88]. Consistently, the comparison of mitochondrial *COI* sequences of a large-scale sample from Japanese and Chinese *B. mandarina* also supported a higher diversity in China compared to Japan [95]. In the case of South Korea, the data of four individuals included in this study are all publicly available. Thus, an additional study with an extended sample size and geographic region is essential for unbiased comparison to other country data. Generally, the maintenance of a large and stable population for long-term biogeographic history is important for the maintenance of genetic diversity [96,97]. In this regard, the Chinese landscape could have been the central region for *B. mandarina* diversity for a long period of time, sustaining larger effective population sizes.

### 4.3. Genetic Relationships of South Korea-Dwelling B. mandarina to Other Regional Populations

Generally speaking, Lepidoptera inhabiting Eastern Asia, including Korea and Japan, are divided into northern and southern species [98,99]. This grouping is set by the Korea Strait, which is a sea passage between the offshore southern tip of South Korea and Tsushima Island in Japan. Thus, northern species are likely to have a distributional range mainly in northeastern Asia, including China to South Korea. Consequently, a closer genetic affinity in the populations living commonly in the Korean peninsula and China, separated from Japan, if not all, could be expected, if no major biogeographic history perturbed such relationships. Indeed, all our analyses strongly indicated a closer genetic relationship of *B. mandarina* in South Korea to those of China, rather than Japan. Thus, our results contradict the previous inference that South Korean *B. mandarina* migrated from Japan throughout the biogeographic event, which occurred during the late glacial era (0.15–0.01 million years ago), when the southwestern areas of Japan were connected to the southern regions of the Korean peninsula [9].

With regard to the progenitor of *B. mori*, Chen et al. [23] first detected the presence of two subtypes in the Chinese *B. mandarina*, each in the southern and northern regions, concordant to the Qinling–Huaihe line, using mitogenome data, and showed that northern Chinese *B. mandarina* are an immediate progenitor for *B. mori* strains. The data obtained in the present study also support those of Chen et al. [23], but our *Nm* estimates (Figure S1), heatmap analysis, based on unrooted pairwise genetic distance (Figure 5) and further sophisticated model-based phylogeny (Figure 6 and Figure S3) with the inclusion of South Korean *B. mandarina*, support that those from both northern China and South Korea are equally the closest group to *B. mori* strains. Furthermore, one *B. mandarina* that originated from Shiquan in China, which is located south of the Qinling–Huaihe line in close proximity (Figure 1), was also grouped together with the northern group in both the heatmap analysis and phylogeny (Figure 5, Figure 6 and Figure S3). Similarly, Sun et al. [22] using the concatenated sequences of *COI* and the A + T-rich region, showed a consistent result that the immediately closer Chinese *B. mandarina* to *B. mori* strains all originated from both the border of the Qinling–Huaihe line (Sichuan and Hubei) and northern China (Shanxi), given that no South Korean samples were included. Considering the vast size and topographical diversity of both northern and southern China, additional sampling could be essential, including of the regions near the Qinling–Huaihe line.

Previously, several studies estimated the divergence time between *B. mori* and *B. mandarina* [9,44,45,100]. Among them, Sun et al. [22] found that domestication of *B. mori* occurred about 4100 before present (BP) with the calibrating point set between 4000 and 11,000 BP based on a fossilized silkworm cocoon shell [13] and silk along with the silk product detected in the prehistoric remains in Chinese villages [101]. This estimation was concordant to the estimation of domestication at 7500–3984 BP, which was calculated under the scenario of the ‘gene flow at bottleneck’ model, using 29 nuclear loci from *B. mandarina* collected from several Chinese regions [102]. Considering that the Yellow Sea now physically separates northern China (north of the Qinling–Huaihe line) and the western part of the Korean peninsula (Figure 1) it is questionable how the *B. mandarina* population dwelling in South Korea could form a panmictic population with those in northern China for at least 4000 years from the time when the domestication of *B. mori* from *B. mandarina* dwelling in northern China occurred.

One plausible explanation could be an extensive gene flow between the northeastern part of China (north of the Qinling–Huaihe line) and west of the Korean peninsula during the last glacial period (some 20,000–12,000 BP), which is often quoted as one of the major events that shaped the current population structure in diverse organisms and regions [103,104,105]. During this period, the Yellow Sea decreased by 120 m to its current level, which is only 44 m on average in depth, with a maximum of 152 m [106], and the lowered sea level connected the western part of the Korean peninsula to the northeastern part of China as plains with rivers. It is likely that during this last glacial period, *B. mandarina* living mainly north of the Qinling–Huaihe line and Korean peninsula may have interacted, resulting in a much closer population constitution than ever before. Moreover, the population increase, particularly during 16,000 and 13,500 YBP, which is close to the end of the Pleistocene, may have further facilitated the dispersal between the two regions (Figure 7). It is worth considering that there are many insect pests, particularly for lepidopteran species, that annually transverse the Yellow Sea currently, dispersing from China to South Korea. These include the tobacco cutworm, *Spodoptera litura*, the rice leaf roller, *Cnaphalocrocis medinalis*, and recently, the fall armyworm, *S. frugiperda*, and they show no genetic divergence between China and South Korea [107,108,109,110,111,112,113]. Although these species could be extreme examples, which have long-distance migratory capacity and dispersal pressure in natal places, they suggest that long-distance dispersal over the Yellow Sea could also be feasible in other lepidopteran species when environmental conditions are favorable for dispersal (e.g., air current and typhoon). Unlike the domesticated *B. mori*, which is unable to fly, *B. mandarina* is a fairly ordinary Lepidoptera for dispersal.

After the Yellow Sea reached the current sea level in ~12,000 BP, two regional populations, nevertheless, unavoidably may have been separated and started to accumulate an independent genetic diversity and consequent genetic divergence. However, gene flow between northeastern China and north of the Korean peninsula could be another route for casual gene flow, considering the current geography of Korea as a peninsula, and this dispersal route could have prevented the genetic separation of *B mandarina* between northern China and South Korea. Given that both samples of *B. mandarina* collected in the vicinity of the northern board of South Korea and southern tip of the Korean peninsula (at least 358 km), respectively, showed no genetic divergence, gene flow through this route could also be effective at preventing the genetic isolation of South Korean populations from northern China, although our sample size is limited and no data from the north of Korea are available. Furthermore, a substantial population increase during 4000 and 2800 YBP, which accompanied warmer climate conditions than before [114,115], may have further facilitated gene flow between the two regions (Figure 7). Indeed, an available example of a butterfly species, named the black-veined white, *Aporia crataegi* (Lepidoptera: Pieridae), which is a northern species distributed throughout most of Europe and temperate Asia to far eastern Russia, Korea, and Japan, evidences such a possibility [116]. Phylogenetic and population genetic analyses using mitochondrial genes (*COI* and *CytB*) and 11 microsatellite loci from the samples collected in South Korea, China (Yanbian Prefecture in Jilin Province), Mongolia, Russia, and Japan showed no genetic divergence and structure among populations, except for that of Japan. Therefore, gene flow directly between the north and south direction over different latitudes could also be important for lepidopteran species, such as *B. mandarina*, which are distributed in the areas concordantly to South Korea and China.

### 4.4. Genetic Relationships of Japan-Dwelling B. mandarina to Other Regional Populations

With regards to the origin of Japanese *B. mandarina*, some of our mitogenome analyses evidenced apomorphic characteristics of Japanese *B. mandarina*, such as a unique repeat sequence in the A + T-rich region (Figure 2) and haplogroup (Figure 3). On the other hand, other analyses showed a closer relationship of Japanese *B. mandarina* to those of southern China, such as the placement of those from Japan as the nearest neighbor to those from southern China in PCoA and heatmap analysis (Figure 4 and Figure 5), and the sister relationship of *B. mandarina* between southern China and Japan in phylogeny (Figure 6 and Figure S3). These results collectively suggest the possibility of *B. mandarina* dwelling in Japan originating from southern China. Consistently, Chen et al. [23] also detected the sister relationship between those from southern China and Japan using mitogenome sequences, given no sample from South Korea.

Previously, several inferences were made with regard to the origin of Japan-dwelling *B. mandarina*, and these include derivation from China, from the Korean peninsula, and artificial introduction to Japan from China, together with *B. mori* [17,21,23,87,117]. Among them, analysis of the *CAD* gene fragment has shown that six of eight sequences from four individuals of *B. mandarina* collected in southern China (Sichuan and Yunnan) clustered together into the same clade with all Japanese *B. mandarina* collected from a diverse region (42 individuals), whereas the remaining sequences of Chinese *B. mandarina*, which were from both northern and southern China, formed another clade together with two haplotypes of *B. mori*, which were obtained from 146 strains native to Japan [87]. Although the study did not include samples from the Korean peninsula, used relatively shorter sequences (~567 bp), and did not show clear grouping, the result is noteworthy in that Chinese *B. mandarina* are divided into two clades: one is immediately close to *B. mori* strains and another, which includes two of the four strains of southern origin, is closer to those of Japan. This result perhaps indicates the involvement of *B. mandarina* in southern China for the origination of *B. mandarina* in Japan, although further extensive study could be essential.

With regard to the time of split between Chinese and Japanese *B. mandarina*, Sun et al. [22] estimated it to be at about 23,600 BP. A possible related geological event could be the complete separation of Japan from the Asian continent, which occurred at about 20,000 BP [118]. If this estimation is applied, *B. mandarina* dwelling in southern China may have arrived in Japan during this time period and started to accumulate independent genetic divergence, resulting in an independent genomic structure in the A + T-rich region and an independent haplogroup (Figure 2, Figure 3, Figure 4, Figure 5 and Figure 6), left as a distant neighbor to those from southern China (Figure 6 and Figure S3).

## 5. Conclusions

In summary, we reported the mitogenome sequences of 37 *B. mori* and four *B. mandarina*, each preserved and collected in South Korea, respectively, and these were combined with 45 preexisting public data entries of the two species. Population genetic analyses and phylogenetic inference confirmed previous views, such as the origin of *B. mori* from Chinese *B. mandarina*, *B. mandarina* dwelling in northern China as an immediate progenitor of *B. mori*, and a divergent genetic relationship of Japanese *B. mandarina* from China. However, our analyses newly detected a closer genetic relationship of South Korean *B. mandarina* to northern China, instead of the long-believed closer relationship to those in Japan. Considering the closer genetic relationship of *B. mandarina* between northern China and South Korea, the potential origin of Japanese *B. mandarina* from southern China is more likely than from any other regions within the present data scope. Although we included 21 *B. mandarina* mitogenome sequences, including four of our own from South Korea, in the analyses, additional study is still required to test some speculation and to overcome the limitation borne from sparse sampling and limited geographic ranges of *B. mandarina*.

## Figures and Tables

**Table 1:** Genetic diversity estimates.

Group	SS ^a^	NH ^b^	*H* ^c^	NP ^d^	MPD ^e^	π ^f^
1. *B. mori* plus *B. mandarina*	86	86	1 ± 0.0018	1985	134.140959 ± 58.074825	0.008199 ± 0.003933
2. *B. mori*	65	65	1 ± 0.0027	615	25.429197 ± 11.302211	0.001603 ± 0.000790
a. South Korea	39	39	1 ± 0.0058	452	16.744353 ± 7.615369	0.001056 ± 0.000534
b. China	16	16	1 ± 0.0221	238	41.835814 ± 19.178396	0.002668 ± 0.001370
c. Japan	4	4	1 ± 0.1768	23	11.841933 ± 6.819167	0.000756 ± 0.000520
d. Europe	3	3	1 ± 0.2722	19	12.674914 ± 7.925171	0.000810 ± 0.000631
e. Cambodia	1	1	1	-	-	-
f. Laos	1	1	1	-	-	-
g. India	1	1	1	-	-	-
3. *B. mandarina*	21	21	1 ± 0.0147	1493	253.292065 ± 112.882473	0.015640 ± 0.007781
a. South Korea	4	4	1 ± 0.1768	160	30.249683 ± 16.885207	0.001923 ± 0.001282
b. China	15	15	1 ± 0.0243	963	185.321893 ± 84.149325	0.011620 ± 0.005916
- southern China	11	11	1 ± 0.0388	234	75.285248 ± 35.188128	0.004800± 0.002531
- northern China	4	4	1 ± 0.1768	333	74.1484056 ± 41.063087	0.004706± 0.003098
c. Japan	2	2	1 ± 0.5000	46	46.125073 ± 32.967009	0.002896 ± 0.002927

^a^ Sample size; ^b^ Number of haplotypes; ^c^ Haplotype diversity with standard error; ^d^ Number of polymorphic sites; ^e^ Mean number of pairwise differences; ^f^ Nucleotide diversity with standard error; -, not available due to a single haplotype.

## Data Availability

The data presented in this study are available in the article and supplementary material here.

## Supplementary Materials

The following are available online at https://www.mdpi.com/article/10.3390/biology11010068/s1, Figure S1. Matrix of migration rate (*Nm*) and genetic distance (*F*_ST_) between pairs of populations, Figure S2: Estimates of pairwise sequence divergence (minimum, average, and maximum), Figure S3: Phylogenetic relationships among *Bombyx mori* and *B. mandarina* haplotypes using the Bayesian inference method, Table S1: List of 88 mitochondrial genomes of *B. mandarina* individuals and *B. mori* strains, including two *Bombyx* species used for data analysis, Table S2: Summary of mitochondrial genomes of 37 *B. mori* strains and four *B. mandarina* individuals obtained in this study, Table S3. Mitochondrial genome characteristics, Table S4: The A + T-rich region sequences of *B. mandarina* reported in Sun et al. [22], Dataset 1. Input files of Arlequin for population analysis, Dataset 2. Xml file of BEAST2 for Skyline plot analysis. References [10,22,23,35,36,37,38,39,40,41,42,43,44,45,46,47,48] are cited in the supplementary materials.
