# Supplementary material for: Phylogeographic Relationships among Bombyx mandarina (Lepidoptera: Bombycidae) Populations and Their Relationships to B. mori Inferred from Mitochondrial Genomes
Source: Biology (Basel). 2022 Jan 3;11(1):68. doi: 10.3390/biology11010068 (PMC8773246; doi:10.3390/biology11010068)
Supplement: Supplementary file 1 [file biology-11-00068-s001.zip › biology-1465686-supplementary.pdf]

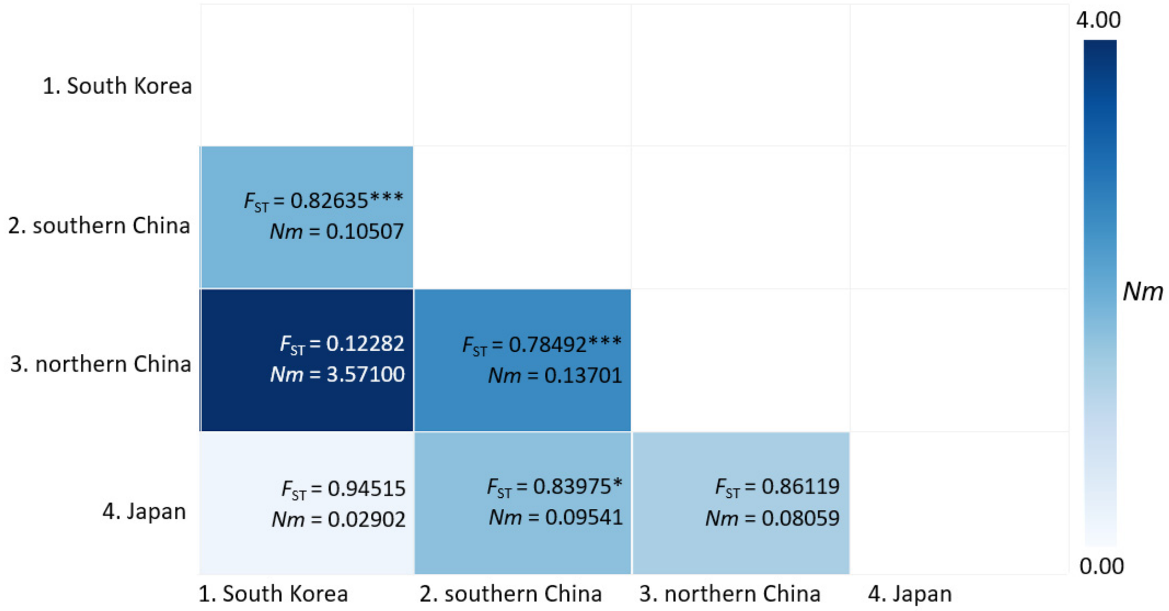

Figure S1. Matrix of migration rate (*Nm*) and genetic distance (F_{ST}) between pairs of populations. *$p < 0.05$, **$p < 0.01$, ***$p < 0.001$.

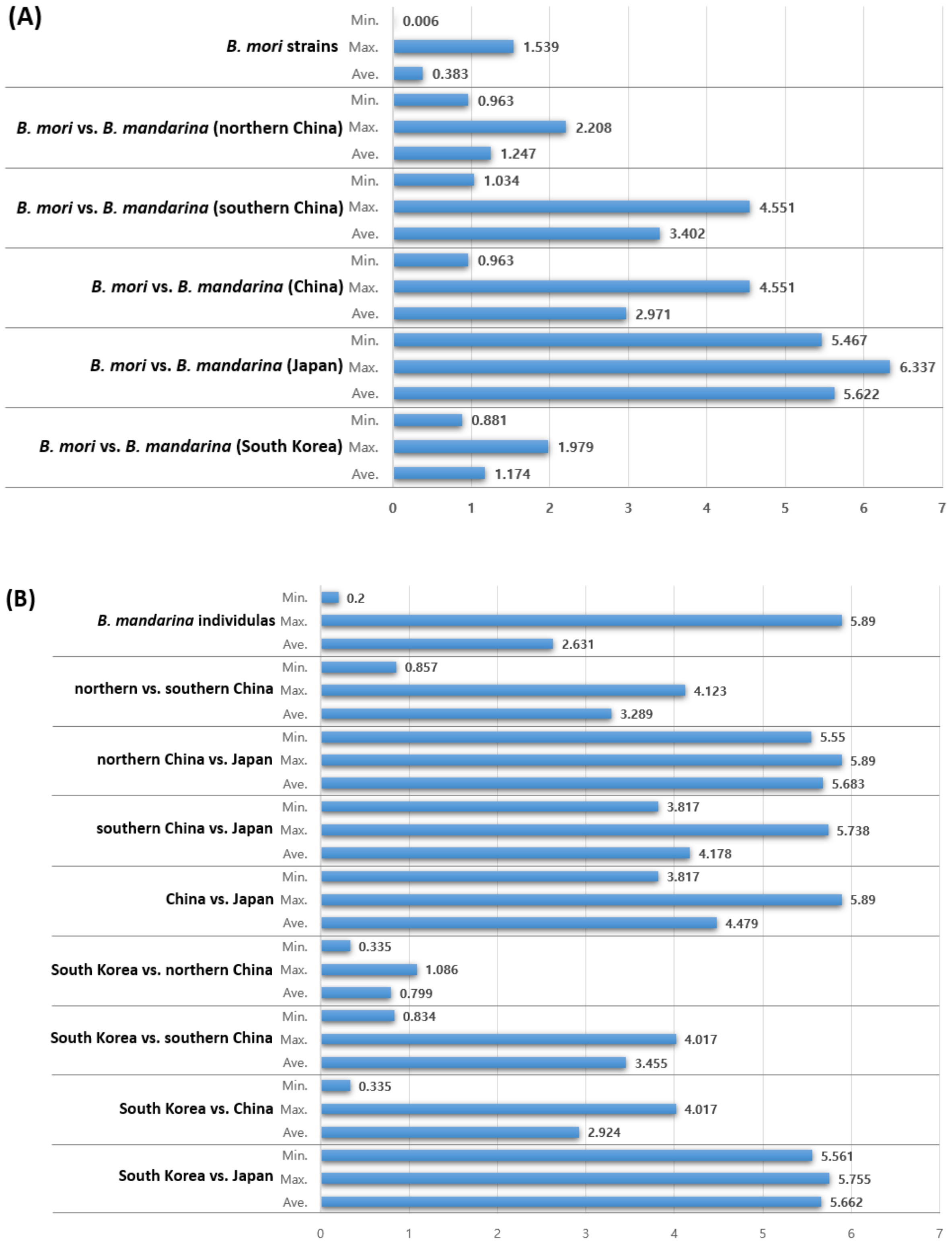

Figure S2. Estimates of pairwise sequence divergence (minimum, average, and maximum). (**A**) Distance between all *Bombyx mori* strains and each *B. mandarina* population. (**B**) Distance between pairs of *B. mandarina* populations.

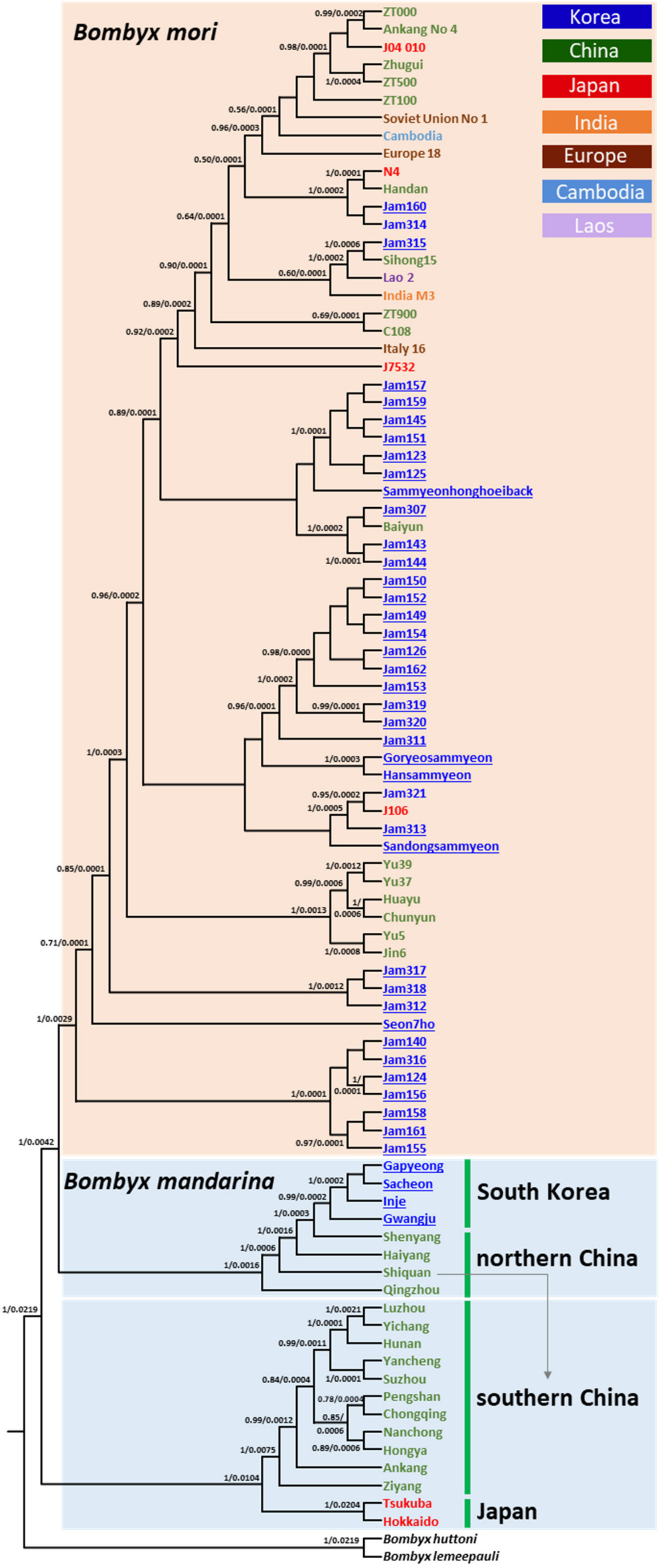

Figure S3. Phylogenetic relationships among *Bombyx mori* and *B. mandarina* haplotypes using the Bayesian inference method. The numbers at each node specify Bayesian posterior

probabilities (BPP; first) and branch length (second). The BPP below 0.5 were omitted. *B. mori* and *B. mandarina* haplotypes sequenced in this study are underlined. *B. huttoni* and *B. lemeepauli* were used as outgroups.

Table S1. List of 88 mitochondrial genomes of *B. mandarina* individuals and *B. mori* strains, including two *Bombyx* species used for data analysis.

No.	Location/Origin of country	Strain/Species	Voucher no.	SRA/GenBank acc. number
***B. mandarina* sequenced in this study**				
1	Gapyeong, South Korea	-	CNU12051	OK358666
2	Inje, South Korea	-	CNU12048	OK358668
3	Gwangju, South Korea	-	CNU14308	OK358667
4	Sacheon, South Korea	-	CNU12047	OK358669
***B. mandarina* obtained from public data**				
5	Tsukuba, Japan	-	-	AB070263
6	Hokkaido, Japan	-	-	GU966593
7	Ziyang, China (southern)	-	-	GU966621
8	Nanchong, China (southern)	-	-	GU966597
9	Hongya, China (southern)	-	-	GU966606
10	Pengshan, China (southern)	-	-	GU966616
11	Luzhou, China (southern)	-	-	GU966631
12	Yancheng, China (southern)	-	-	GU966598
13	Suzhou, China (southern)	-	-	GU966619
14	Yichang, China (southern)	-	-	GU966627
15	Ankang, China (southern)	-	-	GU966591
16	Hunan, China (southern)	-	-	GU966592
17	Chongqing, China (southern)	-	-	GU966629
18	Shiquan, China (southern)	-	-	MN400656
19	Qingzhou, Shandong, China (northern)	-	-	FJ384796
20	Shenyang, Liaoning, China (northern)	-	-	MG604734
21	Haiyang, China (northern)	-	-	MK246423
***B. mori* assembled in this study**				
22	South Korea	Jam123	CNU8256	SRR15338622/ OK358634
23	South Korea	Jam124	CNU8257	SRR15338620/ OK358635
24	South Korea	Jam125	CNU8258	SRR15338621/ OK358636

25	South Korea	Jam126	CNU8259	SRR15338615/ OK358637
26	South Korea	Jam140	CNU8260	SRR15338616/ OK358638
27	South Korea	Jam143	CNU8261	SRR15338617/ OK358639
28	South Korea	Jam144	CNU8262	SRR15338618/ OK358640
29	South Korea	Jam145	CNU8263	SRR15338619/ OK358641
30	South Korea	Jam149	CNU8265	SRR15508057/ OK358642
31	South Korea	Jam150	CNU8266	SRR15508056/ OK358643
32	South Korea	Jam151	CNU8267	SRR15508055/ OK358644
33	South Korea	Jam152	CNU8268	SRR15508054/ OK358645
34	South Korea	Jam153	CNU8269	SRR15514279/ OK358646
35	South Korea	Jam154	CNU8270	SRR15514278/ OK358647
36	South Korea	Jam155	CNU8271	SRR15514277/ OK358648
37	South Korea	Jam156	CNU8272	SRR15514276/ OK358649
38	South Korea	Jam157	CNU8273	SRR15514275/ OK358650
39	South Korea	Jam158	CNU8274	SRR15514274/ OK358651
40	South Korea	Jam159	CNU8275	SRR15514273/ OK358652
41	South Korea	Jam160	CNU8276	SRR15520445/ OK358653
42	South Korea	Jam161	CNU8277	SRR15520444/ OK358654
43	South Korea	Jam162	CNU8278	SRR15520443/ OK358655
44	South Korea	Jam307	CNU8279	SRR15520442/ OK358656
45	South Korea	Jam311	CNU8280	SRR15520441/ OK358657

46	South Korea	Jam312	CNU8281	SRR15520440/ OK358658
47	South Korea	Jam313	CNU8282	SRR15520439/ OK358659
48	South Korea	Jam315	CNU8284	SRR15521832/ OK358660
49	South Korea	Jam316	CNU8285	SRR15521831/ OK358661
50	South Korea	Jam317	CNU8286	SRR15521830/ OK358662
51	South Korea	Jam318	CNU8287	SRR15521829/ OK358663
52	South Korea	Jam319	CNU8288	SRR15521828/ OK358664
53	South Korea	Jam320	CNU8289	SRR15521827/ OK358665
54	South Korea	Goryeosammyeon	CNU8291	SRR15525308/ OK358629
55	South Korea	Sammyeonhonghoeiba ck	CNU8292	SRR15458431/ OK358631
56	South Korea	Hanmammyeon	CNU8293	SRR15458432/ OK358633
57	South Korea	Seon7ho	CNU8294	SRR15458433/ OK358632
58	South Korea	Sandongsammyeon	CNU8295	SRR15458430/ OK358630

***B. mori* obtained from public data**

59	South Korea	Jam314		MK613835
60	South Korea	Jam321		MN103530
61	Japan	J7532	-	GU966620
62	Japan	J04_010	-	GU966612
63	Japan	J106	-	GU966615
64	Japan	N4	-	GU966602
65	Cambodia	Cambodia	-	GU966601
66	Laos	Lao_2	-	GU966610
67	India	India_M3	-	GU966595
68	Europe	Europe_18	-	GU966607
69	Europe	Soviet_Union_No_1	-	GU966599
70	Italy	Italy_16	-	GU966596
71	China	ZT900	-	GU966600
72	China	Zhugui	-	GU966609
73	China	Sihong15	-	GU966617

74	China	ZT500	-	GU966611
75	China	ZT000	-	GU966613
76	China	Ankang_No_4	-	GU966614
77	China	ZT100	-	GU966603
78	China	Handan	-	GU966628
79	China	C108	-	GU966630
80	China	Baiyun	-	KM279431
81	China	Huayu	-	KM875545
82	China	Chunyun	-	KP192478
83	China	Yu5	-	KP192479
84	China	Jin6	-	KP244370
85	China	Yu39	-	KP313778
86	China	Yu37	-	KP729110
***Bombyx* species obtained from public data**				
87	China	*Bombyx huttoni*	-	KP216766
88	China	*Bombyx lemeepauli*	-	KY620270

Sampling location for Chinese *B. mandarina* were divided into northern and southern groups based on the Qinling–Huaihe Line, as shown in Fig. 1. *Bombyx huttoni* and *B. lemeepauli* were mainly used as outgroups for phylogenetic analyses. -, not applicable or not available.

Table S2. The A + T-rich region sequences of *B. mandarina* reported in Sun et al. [22].

No.	Symbol	Location/Origin of country	GenBank acc. number
1	Bma_Y1	Huzhou, Zhejiang, China	GQ423247
2	Bma_Y2	Nanchong, Sichuan, China	GQ423269
3	Bma_Y3	Ankang, Shanxi, China	GQ423271
4	Bma_Y4	Yichang, Hubei, China	GQ423245
5	Bma_Y5	Luzhou, Sichuan, China	GQ423264
6	Bma_Y6	Lixian, Hunan, China	GQ423246
7	Bma_Y7	Suzhou, Jiangsu, Southern China	GQ423248
8	Bma_Y8	Qingmuguan, Chongqing, China	GQ423267
9	Bma_Y9	Rongchang, Chongqing, China	GQ423270
10	Bma_Y11	Ziyang, Sichuan, China	GQ423268
11	Bma_Y13	Hongya, Sichuan, China	GQ423265
12	Bma_Y14	Xiangyun, Yunnan, China	GQ423263
13	Bma_J2	Fukuoka, Japan	GQ423275

14	Bma_J7	Fukuoka, Japan	GQ423274
15	Bma_J227	Fukushima, Japan	GQ423276
16	Bma_J238	Shizuoka, Japan	GQ423272
17	Bma_J341	Hokkaido, Japan	GQ423273

Table S3. Mitochondrial genome characteristics.

Taxon/Strain (*n*)	Origin	M[a]	Size (bp)	A/T content (%)	PCG		*srRNA*		*lrRNA*		tRNA		A+T-rich region		GenBank acc. no.	References
					No. codons[b]	AT (%)	Size (bp)	AT (%)	Size (bp)	AT (%)	Size (bp)	AT (%)	Size (bp)	AT (%)		
***Bombyx mori* (65)**																
Current study (37)																
Goryeosammyeon	Korea	3	15,659	81.37	3,733	79.62	781	85.53	1,376	84.38	1,480	81.76	492	95.33	OK358629	This study
Hansammyeon	Korea	3	15,659	81.37	3,733	79.62	781	85.53	1,378	84.40	1,480	81.76	494	95.34	OK358633	This study
Seon7ho	Korea	3	15,679	81.35	3,733	79.58	781	85.53	1,379	84.34	1,481	81.63	495	95.56	OK358632	This study
Sammyeonhongheoiback	Korea	3	15,652	81.37	3,733	79.63	781	85.53	1,378	84.40	1,480	81.76	494	95.55	OK358631	This study
Sandongsammyeon	Korea	3	15,645	81.35	3,733	79.60	781	85.66	1,377	84.39	1,481	81.77	495	95.56	OK358630	This study
JAM123	Korea	4	15,657	81.36	3,733	79.61	781	85.53	1,379	84.41	1,480	81.76	494	95.55	OK358634	This study
JAM124	Korea	4	15,663	81.34	3,733	79.56	781	85.53	1,378	84.54	1,481	81.63	494	95.55	OK358635	This study
JAM125	Korea	4	15,654	81.36	3,733	79.61	781	85.53	1,379	84.41	1,480	81.76	493	95.54	OK358636	This study
JAM126	Korea	4	15,689	81.41	3,733	79.61	781	85.53	1,377	84.39	1,480	81.76	494	95.55	OK358637	This study
JAM140	Korea	4	15,680	81.36	3,733	79.56	781	85.53	1,378	84.47	1,481	81.63	495	95.56	OK358638	This study
JAM143	Korea	4	15,640	81.33	3,733	79.61	781	85.40	1,378	84.40	1,480	81.76	495	95.56	OK358639	This study
JAM144	Korea	4	15,683	81.39	3,733	79.61	781	85.53	1,377	84.39	1,480	81.76	494	95.55	OK358640	This study
JAM145	Korea	4	15,653	81.36	3,733	79.62	781	85.53	1,378	84.40	1,480	81.76	494	95.55	OK358641	This study
JAM149	Korea	4	15,679	81.40	3,733	79.61	781	85.53	1,377	84.39	1,480	81.76	494	95.55	OK358642	This study
JAM150	Korea	4	15,683	81.40	3,733	79.61	781	85.53	1,377	84.39	1,480	81.76	494	95.55	OK358643	This study
JAM151	Korea	4	15,655	81.36	3,733	79.61	781	85.53	1,378	84.40	1,480	81.76	495	95.56	OK358644	This study
JAM152	Korea	4	15,681	81.40	3,733	79.61	781	85.53	1,377	84.39	1,480	81.76	496	95.56	OK358645	This study
JAM153	Korea	4	15,672	81.39	3,733	79.61	781	85.53	1,377	84.39	1,480	81.69	493	95.54	OK358646	This study
JAM154	Korea	4	15,663	81.38	3,733	79.61	781	85.53	1,377	84.39	1,480	81.76	494	95.55	OK358647	This study
JAM155	Korea	4	15,685	81.36	3,733	79.56	781	85.53	1,378	84.47	1,481	81.63	494	95.55	OK358648	This study
JAM156	Korea	4	15,663	81.34	3,733	79.56	781	85.53	1,378	84.54	1,481	81.63	494	95.55	OK358649	This study
JAM157	Korea	4	15,658	81.36	3,733	79.61	781	85.53	1,378	84.40	1,480	81.76	494	95.55	OK358650	This study
JAM158	Korea	4	15,670	81.35	3,733	79.56	781	85.53	1,378	84.47	1,481	81.63	494	95.55	OK358651	This study
JAM159	Korea	4	15,651	81.36	3,733	79.62	781	85.53	1,379	84.41	1,480	81.76	494	95.55	OK358652	This study
JAM160	Korea	4	15,651	81.36	3,733	79.61	781	85.53	1,378	84.40	1,480	81.82	494	95.55	OK358653	This study

Taxon/Strain (*n*)	Origin	M[a]	Size (bp)	A/T content (%)	PCG		*srRNA*		*lrRNA*		tRNA		A+T-rich region		GenBank acc. no.	References
					No. codons[b]	AT (%)	Size (bp)	AT (%)	Size (bp)	AT (%)	Size (bp)	AT (%)	Size (bp)	AT (%)		
JAM161	Korea	4	15,686	81.37	3,733	79.56	781	85.53	1,378	84.47	1,481	81.63	494	95.55	OK358654	This study
JAM162	Korea	4	15,687	81.40	3,733	79.61	781	85.53	1,377	84.39	1,480	81.76	494	95.55	OK358655	This study
JAM307	Korea	4	15,644	81.35	3,733	79.61	781	85.53	1,378	84.40	1,480	81.69	494	95.55	OK358656	This study
JAM311	Korea	4	15,672	81.40	3,733	79.64	781	85.53	1,377	84.39	1,482	81.71	494	95.55	OK358657	This study
JAM312	Korea	4	15,663	81.36	3,733	79.61	781	85.53	1,377	84.39	1,480	81.62	494	95.34	OK358658	This study
JAM313	S. Korea	4	15,676	81.40	3,733	79.62	781	85.40	1,378	84.40	1,480	81.76	496	95.56	OK358659	This study
JAM315	Korea	4	15,664	81.40	3,733	79.65	781	85.53	1,378	84.47	1,479	81.74	503	95.63	OK358660	This study
JAM316	Korea	4	15,667	81.35	3,733	79.57	781	85.53	1,378	84.47	1,481	81.63	494	95.55	OK358661	This study
JAM317	Korea	4	15,665	81.37	3,733	79.61	781	85.53	1,377	84.39	1,480	81.62	494	95.34	OK358662	This study
JAM318	Korea	4	15,664	81.36	3,733	79.61	781	85.53	1,377	84.39	1,480	81.62	495	95.35	OK358663	This study
JAM319	Korea	4	15,657	81.38	3,733	79.62	781	85.53	1,377	84.39	1,480	81.76	495	95.56	OK358664	This study
JAM320	Korea	4	15,666	81.39	3,733	79.62	781	85.53	1,377	84.39	1,480	81.76	489	95.50	OK358665	This study
GenBank (28)																
JAM314	Korea	4	15,676	81.40	3,733	79.61	781	85.53	1,378	84.40	1,477	81.72	495	95.56	MK613835	Kim et al. [41]
JAM321	Korea	4	15,660	81.39	3,733	79.64	781	85.40	1,378	84.40	1,478	81.73	494	95.55	MN103530	Kim et al. [42]
J7532	Japan	4	15,656	81.35	3,720	79.52	783	85.57	1,378	84.40	1,476	81.64	494	95.55	GU966620	Li et al. [10]
J04-010	Japan	4	15,656	81.35	3,720	79.52	783	85.57	1,378	84.40	1,476	81.57	494	95.55	GU966612	Li et al. [10]
J106	Japan	4	15,656	81.38	3,720	79.56	783	85.44	1,378	84.40	1,476	81.65	494	95.55	GU966615	Li et al. [10]
N4	Japan	4	15,656	81.36	3,720	79.53	783	85.57	1,378	84.40	1,476	81.71	494	95.55	GU966602	Li et al. [10]
Cambodia	Cambodia	4	15,656	81.36	3,720	79.51	783	85.70	1,378	84.54	1,476	81.57	494	95.34	GU966601	Li et al. [10]
Lao 2	Laos	4	15,656	81.38	3,720	79.55	783	85.57	1,378	84.47	1,476	81.64	494	95.55	GU966610	Li et al. [10]
India M3	India	3	15,656	81.36	3,720	79.53	783	85.70	1,378	84.33	1,476	81.64	494	95.55	GU966595	Li et al. [10]
Europe 18	Europe	4	15,656	81.36	3,720	79.53	783	85.57	1,378	84.40	1,476	81.57	494	95.34	GU966607	Li et al. [10]
Italy 16	Italy	4	15,656	81.35	3,720	79.52	783	85.57	1,378	84.40	1,476	81.64	494	95.55	GU966596	Li et al. [10]
Soviet Union No. 1	Europe	4	15,656	81.35	3,720	79.52	783	85.57	1,378	84.40	1,476	81.57	494	95.55	GU966599	Li et al. [10]
ZT900	China	3	15,656	81.36	3,720	79.54	783	85.57	1,378	84.40	1,476	81.72	494	95.55	GU966600	Li et al. [10]
Zhugui	China	4	15,656	81.36	3,720	79.53	783	85.70	1,378	84.40	1,476	81.57	494	95.34	GU966609	Li et al. [10]

Sihong15	China	4	15,656	81.37	3,720	79.54	783	85.57	1,378	84.47	1,476	81.64	494	95.34	GU966617	Li et al. [10]
ZT500	China	3	15,656	81.37	3,720	79.53	783	85.82	1,378	84.40	1,476	81.64	494	95.34	GU966611	Li et al. [10]
ZT000	China	3	15,656	81.33	3,720	79.51	783	85.57	1,378	84.33	1,476	81.50	494	95.55	GU966613	Li et al. [10]
Ankang NO.4	China	3	15,656	81.36	3,720	79.53	783	85.70	1,378	84.40	1,476	81.50	494	95.55	GU966614	Li et al. [10]
ZT100	China	3	15,656	81.35	3,720	79.52	783	85.57	1,378	84.40	1,476	81.58	494	95.55	GU966603	Li et al. [10]
Handan	China	4	15,656	81.37	3,720	79.50	783	85.57	1,378	84.40	1,476	81.72	494	95.55	GU966628	Li et al. [10]
C108	China	4	15,656	81.36	3,720	79.53	783	85.57	1,378	84.40	1,476	81.71	494	95.55	GU966630	Li et al. [10]
Baiyun	China	-	15,629	81.32	3,720	79.53	783	85.57	1,378	84.40	1,473	81.67	496	95.56	KM279431	Zhang et al. [35]
huayu	China	-	15,666	81.33	3,714	79.50	783	85.57	1,378	84.40	1,470	81.50	494	95.55	KM875545	Zhang and Zhou [40]
Chunyun	China	-	15,659	81.36	3,714	79.52	783	85.57	1,379	84.41	1,469	81.55	494	95.34	KP192478	Zhang and Wu [39]
Yu5	China	-	15,644	81.35	3,714	79.51	783	85.57	1,378	84.40	1,468	81.54	494	94.94	KP192479	Zhang et al. [36]
Jin6	China	-	15,648	81.37	3,714	79.51	783	85.57	1,379	84.41	1,468	81.54	494	94.94	KP244370	Zhang et al. [37]
Yu39	China	-	15,652	81.36	3,714	79.47	783	85.57	1,378	84.40	1,470	81.56	494	95.14	KP313778	Zhang et al. [38]
Yu37	China	-	15,658	81.32	3,714	79.52	783	85.57	1,378	84.40	1,470	81.56	494	95.14	KP729110	Zhang and Zhou [40]
**Average**			**15,661**	**81.37**	**3,727**	**79.57**	**782**	**85.55**	**1,378**	**84.41**	**1,478**	**81.67**	**494**	**95.48**		

Taxon/Strain (*n*)	Origin	M[a]	Size (bp)	A/T content (%)	PCG		*srRNA*		*lrRNA*		tRNA		A+T-rich region		GenBank acc. no.	References
					No. codons[b]	AT (%)	Size (bp)	AT (%)	Size (bp)	AT (%)	Size (bp)	AT (%)	Size (bp)	AT (%)		
***Bombyx mandarina* (21)**																
Current study (4)																
	Gapyeong, Korea	3	15,673	81.38	3,733	79.60	780	85.64	1,377	84.60	1,487	81.64	490	95.51	OK358666	This study
	Gwangju, Korea	3	15,701	81.42	3,733	79.59	781	85.79	1,378	84.54	1,487	81.78	493	95.33	OK358667	This study
	Inje, Korea	3	15,657	81.42	3,733	79.59	780	85.77	1,377	84.53	1,488	81.92	485	95.46	OK358668	This study
	Sacheon, Korea	3	15,710	81.44	3,733	79.59	780	85.77	1,379	84.48	1,487	81.84	491	95.52	OK358669	This study
GenBank (17)																
	Tsukuba, Japan	3	15,928	81.68	3,722	79.58	783	85.95	1,377	84.75	1,480	81.55	747	95.18	AB070263	Yukuhiro et al. [44]
	Hokkaido, Japan	3	15,928	81.72	3,722	79.64	783	85.95	1,377	84.68	1,480	81.55	747	95.18	GU966593	Li et al. [10]
	Ziyang, S. China	3	15,682	81.53	3,723	79.76	784	85.46	1,350	84.59	1,488	81.45	484	94.63	GU966621	Li et al. [10]
	Nanchong, S. China	3	15,682	81.47	3,723	79.66	784	85.46	1,350	84.52	1,488	81.59	484	94.83	GU966597	Li et al. [10]
	Hongya, S. China	3	15,682	81.56	3,723	79.77	784	85.46	1,350	84.52	1,488	81.59	484	95.04	GU966606	Li et al. [10]

	Pengshan, S. China	3	15,682	81.48	3,723	79.68	784	85.46	1,350	84.44	1,488	81.52	484	95.04	GU966616	Li et al. [10]
	Luzhou, S. China	3	15,682	81.49	3,723	79.67	784	85.46	1,350	84.59	1,488	81.59	484	94.83	GU966631	Li et al. [10]
	Yancheng, S. China	3	15,682	81.47	3,723	79.66	784	85.33	1,350	84.52	1,488	81.45	484	95.04	GU966598	Li et al. [10]
	Suzhou, S. China	3	15,682	81.50	3,723	79.71	784	85.33	1,350	84.52	1,488	81.65	484	94.42	GU966619	Li et al. [10]
	Yichang, S. China	3	15,682	81.46	3,723	79.66	784	85.46	1,350	84.52	1,488	81.52	484	94.63	GU966627	Li et al. [10]
	Ankang, S. China	3	15,682	81.49	3,723	79.69	784	85.46	1,350	84.52	1,488	81.52	484	94.83	GU966591	Li et al. [10]
	Hunan, S. China	3	15,682	81.51	3,723	79.71	784	85.46	1,350	84.52	1,488	81.45	484	94.63	GU966592	Li et al. [10]
	Chongqing, S. China	3	15,682	81.52	3,723	79.73	784	85.46	1,350	84.44	1,488	81.45	484	95.04	GU966629	Li et al. [10]
	Qingzhou, N. China	3	15,717	81.42	3,714	79.50	788	85.66	1,380	84.64	1,473	81.47	495	95.56	FJ384796	Hu et al. [45]
	Shenyang, N. China	3	15,682	81.44	3,718	79.55	782	85.81	1,377	84.46	1,485	81.82	491	95.52	MG604734	Chen et al. [23]
	Haiyang, N. China	3	15,768	81.45	3,717	79.54	782	85.81	1,378	84.54	1,487	81.84	496	95.56	MK246423	Li et al. [46]
	*Shiquan, S. China	3	15,662	81.46	3,722	79.64	783	85.57	1,379	84.55	1,488	81.52	492	95.53	MN400656	Meng et al. [43]
	**Average**		**15,711**	**81.49**	**3,724**	**79.64**	**783**	**85.60**	**1,363**	**84.55**	**1,486**	**81.61**	**512**	**95.11**		
Bombyx huttoni	China	-	15,638	81.8	3,719	80.04	777	86.74	1,399	85.28	1,476	81.57	512	95.12	KP216766	Peng et al. [47]
Bombyx lemeepauli	China	-	15,801	80.69	3,719	78.61	791	85.84	1,392	83.48	1,490	81.48	764	94.24	KY620270	Liu et al. [48]

[a]M, moltinism.

[b]Termination codons were excluded in the total codon count.

*This genome is registered as a non-annotated format in GenBank, so was annotated in this study.

-, not available.

Table S4. Summary of mitochondrial genomes of 37 *B. mori* strains and four *B. mandarina* individuals obtained in this study.

Gene	Anticodon	Start codon	Stop codon	Goryeosammyeon	Hansammyeon	Seon7ho	Sammyeonhongheoiback	Sandongsammyeon
trnM	CAT			1-68 (68)	1-68 (68)	1-68 (68)	1-68 (68)	1-68 (68)
trnI	GAT			69-131 (63)	69-131 (63)	69-131 (63)	69-131 (63)	69-131 (63)
trnQ	TTG			130-198 (69)	130-198 (69)	130-198 (69)	130-198 (69)	130-198 (69)
ND2		ATA	TAA	246-1268 (1023)	246-1268 (1023)	246-1268 (1023)	246-1268 (1023)	246-1268 (1023)
trnW	TCA			1274-1343 (70)	1274-1343 (70)	1274-1343 (70)	1274-1343 (70)	1274-1343 (70)
trnC	GCA			1336-1402 (67)	1336-1402 (67)	1336-1402 (67)	1336-1402 (67)	1336-1402 (67)
trnY	GTA			1409-1474 (66)	1409-1474 (66)	1409-1474 (66)	1409-1474 (66)	1409-1474 (66)
COI		CGA	T-tRNA	1490-3020 (1531)	1490-3020 (1531)	1490-3020 (1531)	1491-3021 (1531)	1490-3020 (1531)
$trnL_2$	TAA			3021-3087 (67)	3021-3087 (67)	3021-3087 (67)	3022-3088 (67)	3021-3087 (67)
COII		ATG	T-tRNA	3088-3769 (682)	3088-3769 (682)	3088-3769 (682)	3089-3770 (682)	3088-3769 (682)
trnK	CTT			3770-3840 (71)	3770-3840 (71)	3770-3840 (71)	3771-3841 (71)	3770-3840 (71)
trnD	GTC			3840-3906 (67)	3840-3906 (67)	3841-3907 (67)	3841-3907 (67)	3840-3906 (67)
ATP8		ATA	TAA	3907-4068 (162)	3907-4068 (162)	3908-4069 (162)	3908-4069 (162)	3907-4068 (162)
ATP6		ATG	TAA	4062-4739 (678)	4062-4739 (678)	4063-4740 (678)	4063-4740 (678)	4062-4739 (678)
COIII		ATT	TAA	4745-5542 (798)	4745-5542 (798)	4746-5543 (798)	4746-5543 (798)	4745-5542 (798)
trnG	TCC			5545-5610 (66)	5545-5610 (66)	5546-5611 (66)	5546-5611 (66)	5545-5610 (66)
ND3		ATT	TAA	5611-5964 (354)	5611-5964 (354)	5612-5965 (354)	5612-5965 (354)	5611-5964 (354)
trnA	TGC			6015-6082 (68)	6013-6080 (68)	6003-6070 (68)	6006-6073 (68)	6002-6069 (68)
trnR	TCG			6124-6187 (64)	6124-6187 (64)	6114-6177 (64)	6114-6177 (64)	6109-6172 (64)
trnN	GTT			6189-6255 (67)	6189-6255 (67)	6179-6245 (67)	6179-6245 (67)	6174-6240 (67)
$trnS_1$	GCT			6256-6322 (67)	6256-6322 (67)	6246-6312 (67)	6246-6312 (67)	6241-6307 (67)
trnE	TTC			6340-6404 (65)	6340-6404 (65)	6330-6395 (65)	6330-6394 (65)	6325-6389 (65)
trnF	GAA			6404-6470 (67)	6404-6470 (67)	6395-6461 (67)	6394-6460 (67)	6389-6455 (67)
ND5		ATT	TAA	6475-8214 (1740)	6475-8214 (1740)	6466-8205 (1740)	6465-8204 (1740)	6460-8199 (1740)
trnH	GTG			8215-8281 (67)	8215-8281 (67)	8206-8272 (67)	8205-8271 (67)	8200-8266 (67)
ND4		ATT	TAA	8336-9682 (1347)	8334-9680 (1347)	8335-9681 (1347)	8326-9672 (1347)	8319-9665 (1347)
ND4L		ATG	TAA	9676-9966 (291)	9674-9964 (291)	9675-9965 (291)	9666-9956 (291)	9659-9949 (291)
trnT	TGT			9971-10035 (65)	9969-10033 (65)	9970-10034 (65)	9961-10025 (65)	9954-10018 (65)
trnP	TGG			10036-10101 (66)	10034-10099 (66)	10035-10100 (66)	10026-10091 (66)	10019-10085 (67)
ND6		ATT	TAA	10104-10634 (531)	10102-10632 (531)	10103-10633 (531)	10094-10624 (531)	10088-10618 (531)
CytB		ATG[1]/ATC[2]	TAA	10677-11834 (1158)[1]	10675-11832 (1158)[1]	10693-11850 (1158)[2]	10668-11825 (1158)[1]	10661-11818 (1158)[1]
$trnS_2$	TGA			11837-11904 (68)	11835-11902 (68)	11853-11920 (68)	11828-11895 (68)	11821-11888 (68)
ND1		ATT	TAA	11929-12867 (939)	11927-12865 (939)	11945-12883 (939)	11920-12858 (939)	11913-12851 (939)

trnL$_1$	TAG	12868-12938 (71)	12866-12936 (71)	12884-12954 (71)	12859-12929 (71)	12852-12922 (71)
lrRNA		12939-14316 (1378)	12937-14314 (1378)	12955-14333 (1379)	12930-14307 (1378)	12923-14299 (1377)
trnV	TAC	14317-14384 (68)	14315-14382 (68)	14334-14401 (68)	14308-14375 (68)	14300-14367 (68)
srRNA		14387-15167 (781)	14385-15165 (781)	14404-15184 (781)	14378-15158 (781)	14370-15150 (781)
A+T–rich region		15168-15659 (492)	15166-15659 (494)	15185-15679 (495)	15159-15652 (494)	15151-15645 (495)

Gene	Anticodon	Start codon	Stop codon	Jam123	Jam124	Jam125	Jam126	Jam140	Jam143
trnM	CAT			1-68 (68)	1-68 (68)	1-68 (68)	1-68 (68)	1-68 (68)	1-68 (68)
trnI	GAT			69-131 (63)	69-131 (63)	69-131 (63)	69-131 (63)	69-131 (63)	69-131 (63)
trnQ	TTG			130-198 (69)	130-198 (69)	130-198 (69)	130-198 (69)	130-198 (69)	130-198 (69)
ND2		ATA	TAA	246-1268 (1023)	246-1268 (1023)	246-1268 (1023)	246-1268 (1023)	246-1268 (1023)	246-1268 (1023)
trnW	TCA			1274-1343 (70)	1274-1343 (70)	1274-1343 (70)	1274-1343 (70)	1274-1343 (70)	1274-1343 (70)
trnC	GCA			1336-1402 (67)	1336-1402 (67)	1336-1402 (67)	1336-1402 (67)	1336-1402 (67)	1336-1402 (67)
trnY	GTA			1409-1474 (66)	1409-1474 (66)	1409-1474 (66)	1409-1474 (66)	1409-1474 (66)	1409-1474 (66)
COI		CGA	T-tRNA	1490-3020 (1531)	1491-3021 (1531)	1490-3020 (1531)	1491-3021 (1531)	1491-3021 (1531)	1490-3020 (1531)
trnL$_2$	TAA			3021-3087 (67)	3022-3088 (67)	3021-3087 (67)	3022-3088 (67)	3022-3088 (67)	3021-3087 (67)
COII		ATG	T-tRNA	3088-3769 (682)	3089-3770 (682)	3088-3769 (682)	3089-3770 (682)	3089-3770 (682)	3088-3769 (682)
trnK	CTT			3770-3840 (71)	3771-3841 (71)	3770-3840 (71)	3771-3841 (71)	3771-3841 (71)	3770-3840 (71)
trnD	GTC			3840-3906 (67)	3841-3907 (67)	3840-3906 (67)	3841-3907 (67)	3841-3907 (67)	3840-3906 (67)
ATP8		ATA	TAA	3907-4068 (162)	3908-4069 (162)	3907-4068 (162)	3908-4069 (162)	3908-4069 (162)	3907-4068 (162)
ATP6		ATG	TAA	4062-4739 (678)	4063-4740 (678)	4062-4739 (678)	4063-4740 (678)	4063-4740 (678)	4062-4739 (678)
COIII		ATT	TAA	4745-5542 (798)	4746-5543 (798)	4745-5542 (798)	4746-5543 (798)	4746-5543 (798)	4745-5542 (798)
trnG	TCC			5545-5610 (66)	5546-5611 (66)	5545-5610 (66)	5546-5611 (66)	5546-5611 (66)	5545-5610 (66)
ND3		ATT	TAA	5611-5964 (354)	5612-5965 (354)	5611-5964 (354)	5612-5965 (354)	5612-5965 (354)	5611-5964 (354)
trnA	TGC			5999-6066 (68)	6008-6075 (68)	5999-6066 (68)	6028-6095 (68)	6024-6091 (68)	5994-6061 (68)
trnR	TCG			6113-6176 (64)	6113-6176 (64)	6113-6176 (64)	6155-6218 (64)	6133-6196 (64)	6096-6159 (64)
trnN	GTT			6178-6244 (67)	6178-6244 (67)	6178-6244 (67)	6220-6286 (67)	6198-6264 (67)	6161-6227 (67)
trnS$_1$	GCT			6245-6311 (67)	6245-6311 (67)	6245-6311 (67)	6287-6353 (67)	6265-6331 (67)	6228-6294 (67)
trnE	TTC			6329-6393 (65)	6329-6394 (66)	6329-6393 (65)	6371-6435 (65)	6349-6414 (66)	6312-6376 (65)
trnF	GAA			6393-6459 (67)	6394-6460 (67)	6393-6459 (67)	6435-6501 (67)	6414-6480 (67)	6376-6442 (67)
ND5		ATT	TAA	6464-8203 (1740)	6465-8204 (1740)	6464-8203 (1740)	6506-8245 (1740)	6485-8224 (1740)	6447-8186 (1740)
trnH	GTG			8204-8270 (67)	8205-8271 (67)	8204-8270 (67)	8246-8312 (67)	8225-8291 (67)	8187-8253 (67)
ND4		ATT	TAA	8331-9677 (1347)	8336-9682 (1347)	8329-9675 (1347)	8366-9712 (1347)	8352-9698 (1347)	8314-9660 (1347)
ND4L		ATG	TAA	9671-9961 (291)	9676-9966 (291)	9669-9959 (291)	9706-9996 (291)	9692-9982 (291)	9654-9944 (291)

trnT	TGT			9966-10030 (65)	9971-10035 (65)	9964-10028 (65)	10001-10065 (65)	9987-10051 (65)	9949-10013 (65)
trnP	TGG			10031-10096 (66)	10036-10101 (66)	10029-10094 (66)	10066-10131 (66)	10052-10117 (66)	10014-10079 (66)
ND6		ATT	TAA	10099-10629 (531)	10104-10634 (531)	10097-10627 (531)	10134-10664 (531)	10120-10650 (531)	10082-10612 (531)
CytB		ATG[1]/ATC[2]	TAA	10672-11829 (1158)[1]	10679-11836 (1158)[1]	10670-11827 (1158)[1]	10706-11863 (1158)[1]	10695-11852 (1158)[1]	10655-11812 (1158)[1]
$trnS_2$	TGA			11832-11899 (68)	11839-11906 (68)	11830-11897 (68)	11866-11933 (68)	11855-11922 (68)	11815-11882 (68)
ND1		ATT	TAA	11924-12862 (939)	11931-12869 (939)	11922-12860 (939)	11958-12896 (939)	11947-12885 (939)	11907-12845 (939)
$trnL_1$	TAG			12863-12933 (71)	12870-12940 (71)	12861-12931 (71)	12897-12967 (71)	12886-12956 (71)	12846-12916 (71)
lrRNA				12934-14312 (1379)	12941-14318 (1378)	12932-14310 (1379)	12968-14344 (1377)	12957-14312 (1378)	12917-14294 (1378)
trnV	TAC			14313-14380 (68)	14319-14386 (68)	14311-14378 (68)	14345-14412 (68)	14335-14402 (68)	14295-14362 (68)
srRNA				14383-15163 (781)	14389-15169 (781)	14381-15161 (781)	14415-15195 (781)	14405-15185 (781)	14365-15145 (781)
A+T–rich region				15164-15657 (494)	15170-15663 (494)	15162-15654 (493)	15196-15689 (494)	15186-15680 (495)	15146-15640 (495)

Gene	Anticodon	Start codon	Stop codon	Jam144	Jam145	Jam149	Jam150	Jam151	Jam152
trnM	CAT			1-68 (68)	1-68 (68)	1-68 (68)	1-68 (68)	1-68 (68)	1-68 (68)
trnI	GAT			69-131 (63)	69-131 (63)	69-131 (63)	69-131 (63)	69-131 (63)	69-131 (63)
trnQ	TTG			130-198 (69)	130-198 (69)	130-198 (69)	130-198 (69)	130-198 (69)	130-198 (69)
ND2		ATA	TAA	246-1268 (1023)	246-1268 (1023)	246-1268 (1023)	246-1268 (1023)	246-1268 (1023)	246-1268 (1023)
trnW	TCA			1274-1343 (70)	1274-1343 (70)	1274-1343 (70)	1274-1343 (70)	1274-1343 (70)	1274-1343 (70)
trnC	GCA			1336-1402 (67)	1336-1402 (67)	1336-1402 (67)	1336-1402 (67)	1336-1402 (67)	1336-1402 (67)
trnY	GTA			1409-1474 (66)	1409-1474 (66)	1409-1474 (66)	1409-1474 (66)	1409-1474 (66)	1409-1474 (66)
COI		CGA	T-tRNA	1491-3021 (1531)	1490-3020 (1531)	1491-3021 (1531)	1491-3021 (1531)	1490-3020 (1531)	1491-3021 (1531)
$trnL_2$	TAA			3022-3088 (67)	3021-3087 (67)	3022-3088 (67)	3022-3088 (67)	3021-3087 (67)	3022-3088 (67)
COII		ATG	T-tRNA	3089-3770 (682)	3088-3769 (682)	3089-3770 (682)	3089-3770 (682)	3088-3769 (682)	3089-3770 (682)
trnK	CTT			3771-3841 (71)	3770-3840 (71)	3771-3841 (71)	3771-3841 (71)	3770-3840 (71)	3771-3841 (71)
trnD	GTC			3841-3907 (67)	3840-3906 (67)	3841-3907 (67)	3841-3907 (67)	3840-3906 (67)	3841-3907 (67)
ATP8		ATA	TAA	3908-4069 (162)	3907-4068 (162)	3908-4069 (162)	3908-4069 (162)	3907-4068 (162)	3908-4069 (162)
ATP6		ATG	TAA	4063-4740 (678)	4062-4739 (678)	4063-4740 (678)	4063-4740 (678)	4062-4739 (678)	4063-4740 (678)
COIII		ATT	TAA	4746-5543 (798)	4745-5542 (798)	4746-5543 (798)	4746-5543 (798)	4745-5542 (798)	4746-5543 (798)
trnG	TCC			5546-5611 (66)	5545-5610 (66)	5546-5611 (66)	5546-5611 (66)	5545-5610 (66)	5546-5611 (66)
ND3		ATT	TAA	5612-5965 (354)	5611-5964 (354)	5612-5965 (354)	5612-5965 (354)	5611-5964 (354)	5612-5965 (354)
trnA	TGC			6034-6101 (68)	6002-6069 (68)	6028-6095 (68)	6026-6093 (68)	6007-6074 (68)	6032-6099 (68)
trnR	TCG			6153-6216 (64)	6112-6175 (64)	6147-6210 (64)	6151-6214 (64)	6117-6180 (64)	6149-6212 (64)

trnN	GTT			6218-6284 (67)	6177-6243 (67)	6212-6278 (67)	6216-6282 (67)	6182-6248 (67)	6214-6280 (67)
$trnS_1$	GCT			6285-6351 (67)	6244-6310 (67)	6279-6345 (67)	6283-6349 (67)	6249-6315 (67)	6281-6347 (67)
trnE	TTC			6369-6433 (65)	6328-6392 (65)	6363-6427 (65)	6367-6431 (65)	6333-6397 (65)	6365-6429 (65)
trnF	GAA			6433-6499 (67)	6392-6458 (67)	6427-6493 (67)	6431-6497 (67)	6397-6463 (67)	6429-6495 (67)
ND5		ATT	TAA	6504-8243 (1740)	6463-8202 (1740)	6498-8237 (1740)	6502-8241 (1740)	6468-8207 (1740)	6500-8239 (1740)
trnH	GTG			8244-8310 (67)	8203-8269 (67)	8238-8304 (67)	8242-8308 (67)	8208-8274 (67)	8240-8306 (67)
ND4		ATT	TAA	8360-9706 (1347)	8328-9674 (1347)	8356-9702 (1347)	8360-9706 (1347)	8329-9675 (1347)	8356-9702 (1347)
ND4L		ATG	TAA	9700-9990 (291)	9668-9958 (291)	9696-9986 (291)	9700-9990 (291)	9669-9959 (291)	9696-9986 (291)
trnT	TGT			9995-10059 (65)	9963-10027 (65)	9991-10055 (65)	9995-10059 (65)	9964-10028 (65)	9991-10055 (65)
trnP	TGG			10060-10125 (66)	10028-10093 (66)	10056-10121 (66)	10060-10125 (66)	10029-10094 (66)	10056-10121 (66)
ND6		ATT	TAA	10128-10658 (531)	10096-10626 (531)	10124-10654 (531)	10128-10658 (531)	10097-10627 (531)	10124-10654 (531)
CytB		ATG[1]/ATC[2]	TAA	10700-11857 (1158)[1]	10669-11826 (1158)[1]	10696-11853 (1158)[1]	10700-11857 (1158)[1]	10670-11827 (1158)[1]	10696-11853 (1158)[1]
$trnS_2$	TGA			11860-11927 (68)	11829-11896 (68)	11856-11923 (68)	11860-11927 (68)	11830-11897 (68)	11856-11923 (68)
ND1		ATT	TAA	11952-12890 (939)	11921-12859 (939)	11948-12886 (939)	11952-12890 (939)	11922-12860 (939)	11948-12886 (939)
$trnL_1$	TAG			12891-12961 (71)	12860-12930 (71)	12887-12957 (71)	12891-12961 (71)	12861-12931 (71)	12887-12957 (71)
lrRNA				12962-14338 (1377)	12931-14308 (1378)	12958-14334 (1377)	12962-14338 (1377)	12932-14309 (1378)	12958-14334 (1377)
trnV	TAC			14339-14406 (68)	14309-14376 (68)	14335-14402 (68)	14339-14406 (68)	14377-14310 (68)	14335-14402 (68)
srRNA				14409-15189 (781)	14379-15159 (781)	14405-15185 (781)	14409-15189 (781)	14380-15160 (781)	14405-15185 (781)
A+T–rich region				15190-15683 (494)	15160-15653 (494)	15186-15679 (494)	15190-15683 (494)	15161-15655 (495)	15186-15681 (496)

Gene	Anticodon	Start codon	Stop codon	Jam153	Jam154	Jam155	Jam156	Jam157	Jam158
trnM	CAT			1-68 (68)	1-68 (68)	1-68 (68)	1-68 (68)	1-68 (68)	1-68 (68)
trnI	GAT			69-131 (63)	69-131 (63)	69-131 (63)	69-131 (63)	69-131 (63)	69-131 (63)
trnQ	TTG			130-198 (69)	130-198 (69)	130-198 (69)	130-198 (69)	130-198 (69)	130-198 (69)
ND2		ATA	TAA	246-1268 (1023)	246-1268 (1023)	246-1268 (1023)	246-1268 (1023)	246-1268 (1023)	246-1268 (1023)
trnW	TCA			1274-1343 (70)	1274-1343 (70)	1274-1343 (70)	1274-1343 (70)	1274-1343 (70)	1274-1343 (70)
trnC	GCA			1336-1402 (67)	1336-1402 (67)	1336-1402 (67)	1336-1402 (67)	1336-1402 (67)	1336-1402 (67)
trnY	GTA			1409-1474 (66)	1409-1474 (66)	1409-1474 (66)	1409-1474 (66)	1409-1474 (66)	1409-1474 (66)
COI		CGA	T-tRNA	1491-3021 (1531)	1491-3021 (1531)	1491-3021 (1531)	1491-3021 (1531)	1490-3020 (1531)	1491-3021 (1531)
$trnL_2$	TAA			3022-3088 (67)	3022-3088 (67)	3022-3088 (67)	3022-3088 (67)	3021-3087 (67)	3022-3088 (67)
COII		ATG	T-tRNA	3089-3770 (682)	3089-3770 (682)	3089-3770 (682)	3089-3770 (682)	3088-3769 (682)	3089-3770 (682)
trnK	CTT			3771-3841 (71)	3771-3841 (71)	3771-3841 (71)	3771-3841 (71)	3770-3840 (71)	3771-3841 (71)

trnD	GTC			3841-3907 (67)	3841-3907 (67)	3841-3907 (67)	3841-3907 (67)	3840-3906 (67)	3841-3907 (67)
ATP8		ATA	TAA	3908-4069 (162)	3908-4069 (162)	3908-4069 (162)	3908-4069 (162)	3907-4068 (162)	3908-4069 (162)
ATP6		ATG	TAA	4063-4740 (678)	4063-4740 (678)	4063-4740 (678)	4063-4740 (678)	4062-4739 (678)	4063-4740 (678)
COIII		ATT	TAA	4746-5543 (798)	4746-5543 (798)	4746-5543 (798)	4746-5543 (798)	4745-5542 (798)	4746-5543 (798)
trnG	TCC			5546-5611 (66)	5546-5611 (66)	5546-5611 (66)	5546-5611 (66)	5545-5610 (66)	5546-5611 (66)
ND3		ATT	TAA	5612-5965 (354)	5612-5965 (354)	5612-5965 (354)	5612-5965 (354)	5611-5964 (354)	5612-5965 (354)
trnA	TGC			6020-6087 (68)	6006-6073 (68)	6024-6091 (68)	6008-6075 (68)	6005-6072 (68)	6017-6084 (68)
trnR	TCG			6141-6204 (64)	6131-6194 (64)	6145-6208 (64)	6115-6178 (64)	6117-6180 (64)	6130-6193 (64)
trnN	GTT			6206-6272 (67)	6196-6262 (67)	6210-6276 (67)	6180-6246 (67)	6182-6248 (67)	6195-6261 (67)
trnS$_1$	GCT			6273-6339 (67)	6263-6329 (67)	6277-6343 (67)	6247-6313 (67)	6249-6315 (67)	6262-6328 (67)
trnE	TTC			6357-6421 (65)	6347-6411 (65)	6361-6426 (65)	6331-6396 (66)	6333-6397 (65)	6346-6397 (66)
trnF	GAA			6421-6487 (67)	6411-6482 (67)	6426-6492 (67)	6396-6462 (67)	6397-6463 (67)	6397-6463 (67)
ND5		ATT	TAA	6492-8231 (1740)	6482-8221 (1740)	6497-8236 (1740)	6467-8206 (1740)	6468-8207 (1740)	6482-8221 (1740)
trnH	GTG			8232-8298 (67)	8222-8288 (67)	8237-8303 (67)	8207-8273 (67)	8208-8274 (67)	8222-8288 (67)
ND4		ATT	TAA	8350-9696 (1347)	8340-9686 (1347)	8358-9704 (1347)	8336-9682 (1347)	8333-9679 (1347)	8343-9689 (1347)
ND4L		ATG	TAA	9690-9980 (291)	9680-9970 (291)	9698-9988 (291)	9676-9966 (291)	9673-9963 (291)	9683-9973 (291)
trnT	TGT			9985-10049 (65)	9975-10039 (65)	9993-10057 (65)	9971-10035 (65)	9968-10032 (65)	9978-10042 (65)
trnP	TGG			10050-10115 (66)	10040-10105 (66)	10058-10123 (66)	10036-10101 (66)	10033-10098 (66)	10043-10108 (66)
ND6		ATT	TAA	10118-10648 (531)	10108-10638 (531)	10126-10656 (531)	10104-10634 (531)	10101-10631 (531)	10111-10641 (531)
CytB		ATG[1]/ATC[2]	TAA	10690-11847 (1158)[1]	10680-11837 (1158)[1]	10701-11858 (1158)[1]	10679-11836 (1158)[1]	10674-11831 (1158)[1]	10686-11843 (1158)[1]
trnS$_2$	TGA			11850-11917 (68)	11840-11907 (68)	11861-11928 (68)	11839-11906 (68)	11834-11901 (68)	11846-11913 (68)
ND1		ATT	TAA	11942-12880 (939)	11932-12870 (939)	11953-12891 (939)	11931-12869 (939)	11926-12864 (939)	11938-12876 (939)
trnL$_1$	TAG			12881-12951 (71)	12871-12941 (71)	12892-12962 (71)	12870-12940 (71)	12865-12935 (71)	12877-12947 (71)
lrRNA				12952-14328 (1377)	12942-14318 (1377)	12963-14340 (1378)	12941-14318 (1378)	12936-14313 (1378)	12948-14325 (1378)
trnV	TAC			14329-14396 (68)	14319-14386 (68)	14341-14408 (68)	14319-14386 (68)	14314-14381 (68)	14326-14393 (68)
srRNA				14399-15179 (781)	14389-15169 (781)	14411-15191 (781)	14389-15169 (781)	14384-15164 (781)	143963-15176 (781)
A+T–rich region				15180-15672 (493)	15170-15663 (494)	15192-15685 (494)	15170-15663 (494)	15165-15658 (494)	15177-15670 (494)

Gene	Anticodon	Start codon	Stop codon	Jam159	Jam160	Jam161	Jam162	Jam307	Jam311
trnM	CAT			1-68 (68)	1-68 (68)	1-68 (68)	1-68 (68)	1-68 (68)	1-68 (68)
trnI	GAT			69-131 (63)	69-131 (63)	69-131 (63)	69-131 (63)	69-131 (63)	69-131 (63)
trnQ	TTG			130-198 (69)	130-198 (69)	130-198 (69)	130-198 (69)	130-198 (69)	130-198 (69)

ND2		ATA	TAA	246-1268 (1023)	246-1268 (1023)	246-1268 (1023)	246-1268 (1023)	246-1268 (1023)	246-1268 (1023)
trnW	TCA			1274-1343 (70)	1274-1343 (70)	1274-1343 (70)	1274-1343 (70)	1274-1343 (70)	1274-1343 (70)
trnC	GCA			1336-1402 (67)	1336-1402 (67)	1336-1402 (67)	1336-1402 (67)	1336-1402 (67)	1336-1402 (67)
trnY	GTA			1409-1474 (66)	1409-1474 (66)	1409-1474 (66)	1409-1474 (66)	1409-1474 (66)	1409-1474 (66)
COI		CGA	T-tRNA	1490-3020 (1531)	1490-3020 (1531)	1491-3021 (1531)	1491-3021 (1531)	1490-3020 (1531)	1490-3020 (1531)
trnL$_2$	TAA			3021-3087 (67)	3021-3087 (67)	3022-3088 (67)	3022-3088 (67)	3021-3087 (67)	3021-3087 (67)
COII		ATG	T-tRNA	3088-3769 (682)	3088-3769 (682)	3089-3770 (682)	3089-3770 (682)	3088-3769 (682)	3088-3769 (682)
trnK	CTT			3770-3840 (71)	3770-3840 (71)	3771-3841 (71)	3771-3841 (71)	3770-3840 (71)	3770-3840 (71)
trnD	GTC			3840-3906 (67)	3840-3906 (67)	3841-3907 (67)	3841-3907 (67)	3840-3906 (67)	3840-3906 (67)
ATP8		ATA	TAA	3907-4068 (162)	3907-4068 (162)	3908-4069 (162)	3908-4069 (162)	3907-4068 (162)	3907-4068 (162)
ATP6		ATG	TAA	4062-4739 (678)	4062-4739 (678)	4063-4740 (678)	4063-4740 (678)	4062-4739 (678)	4062-4739 (678)
COIII		ATT	TAA	4745-5542 (798)	4745-5542 (798)	4746-5543 (798)	4746-5543 (798)	4745-5542 (798)	4745-5542 (798)
trnG	TCC			5545-5610 (66)	5545-5610 (66)	5546-5611 (66)	5546-5611 (66)	5545-5610 (66)	5545-5610 (66)
ND3		ATT	TAA	5611-5964 (354)	5611-5964 (354)	5612-5965 (354)	5612-5965 (354)	5611-5964 (354)	5611-5964 (354)
trnA	TGC			6001-6068 (68)	6009-6076 (68)	6025-6092 (68)	6026-6093 (68)	5996-6063 (68)	6035-6102 (68)
trnR	TCG			6109-6172 (64)	6110-6173 (64)	6146-6209 (64)	6153-6216 (64)	6104-6167 (64)	6140-6203 (64)
trnN	GTT			6174-6240 (67)	6175-6241 (67)	6211-6277 (67)	6218-6284 (67)	6169-6235 (67)	6205-6271 (67)
trnS$_1$	GCT			6241-6307 (67)	6242-6308 (67)	6278-6344 (67)	6285-6351 (67)	6236-6302 (67)	6272-6338 (67)
trnE	TTC			6325-6389 (65)	6326-6390 (65)	6247-6362 (66)	6369-6433 (65)	6320-6384 (65)	6356-6422 (67)
trnF	GAA			6389-6325 (67)	6390-6456 (67)	6427-6493 (67)	6433-6499 (67)	6384-6450 (67)	6422-6488 (67)
ND5		ATT	TAA	6460-8199 (1740)	6461-8200 (1740)	6498-8237 (1740)	6504-8243 (1740)	6455-8194 (1740)	6493-8232 (1740)
trnH	GTG			8200-8266 (67)	8201-8267 (67)	8238-8304 (67)	8244-8310 (67)	8195-8261 (67)	8233-8299 (67)
ND4		ATT	TAA	8325-9671 (1347)	8326-9672 (1347)	8359-9705 (1347)	8364-9710 (1347)	8319-9665 (1347)	8346-9692 (1347)
ND4L		ATG	TAA	9665-9955 (291)	9666-9956 (291)	9699-9989 (291)	9704-9994 (291)	9659-9949 (291)	9686-9976 (291)
trnT	TGT			9960-10024 (65)	9961-10025 (65)	9994-10058 (65)	9999-10063 (65)	9954-10018 (65)	9981-10045 (65)
trnP	TGG			10025-10090 (66)	10026-10091 (66)	10059-10124 (66)	10064-10129 (66)	10019-10084 (66)	10045-10111 (66)
ND6		ATT	TAA	10093-10623 (531)	10094-10624 (531)	10127-10657 (531)	10132-10662 (531)	10087-10617 (531)	10114-10644 (531)
CytB		ATG[1]/ATC[2]	TAA	10666-11823 (1158)[1]	10667-11824 (1158)[1]	10702-11859 (1158)[1]	10704-11861 (1158)[1]	10660-11817 (1158)[1]	10689-11846 (1158)[1]
trnS$_2$	TGA			11826-11893 (68)	11827-11894 (68)	11862-11929 (68)	11864-11931 (68)	11820-11887 (68)	11849-11916 (68)
ND1		ATT	TAA	11918-12856 (939)	11919-12857 (939)	11954-12892 (939)	11956-12894 (939)	11912-12850 (939)	11941-12879 (939)
trnL$_1$	TAG			12857-12927 (71)	12858-12928 (71)	12893-12963 (71)	12895-12965 (71)	12851-12921 (71)	12880-12950 (71)
lrRNA				12928-14306 (1379)	12929-14306 (1378)	12964-14341 (1378)	12966-14342 (1377)	12922-14299 (1378)	12951-14327 (1377)
trnV	TAC			14307-14374 (68)	14307-14374 (68)	14342-14409 (68)	14343-14410 (68)	14300-14367 (68)	14328-14395 (68)
srRNA				14377-15157 (781)	14377-15157 (781)	14412-15192 (781)	14413-15193 (781)	14370-15150 (781)	14938-15178 (781)

A+T–rich region				15158-15651 (494)	15158-15651 (494)	15193-15686 (494)	15194-15687 (494)	15151-15644 (494)	15179-15672 (494)

Gene	Anticodon	Start codon	Stop codon	Jam312	Jam313	Jam315	Jam316	Jam317	Jam318
trnM	CAT			1-68 (68)	1-68 (68)	1-68 (68)	1-68 (68)	1-68 (68)	1-68 (68)
trnI	GAT			69-131 (63)	69-131 (63)	69-131 (63)	69-131 (63)	69-131 (63)	69-131 (63)
trnQ	TTG			130-198 (69)	130-198 (69)	130-198 (69)	130-198 (69)	130-198 (69)	130-198 (69)
ND2		ATA	TAA	246-1268 (1023)	246-1268 (1023)	246-1268 (1023)	246-1268 (1023)	246-1268 (1023)	246-1268 (1023)
trnW	TCA			1274-1343 (70)	1274-1343 (70)	1274-1343 (70)	1274-1343 (70)	1274-1343 (70)	1274-1343 (70)
trnC	GCA			1336-1402 (67)	1336-1402 (67)	1336-1402 (67)	1336-1402 (67)	1336-1402 (67)	1336-1402 (67)
trnY	GTA			1409-1474 (66)	1409-1474 (66)	1409-1474 (66)	1409-1474 (66)	1409-1474 (66)	1409-1474 (66)
COI		CGA	T-tRNA	1491-3021 (1531)	1490-3020 (1531)	1490-3020 (1531)	1491-3021 (1531)	1491-3021 (1531)	1491-3021 (1531)
trnL$_2$	TAA			3022-3088 (67)	3021-3087 (67)	3021-3087 (67)	3022-3088 (67)	3022-3088 (67)	3022-3088 (67)
COII		ATG	T-tRNA	3089-3770 (682)	3088-3769 (682)	3088-3769 (682)	3089-3770 (682)	3089-3770 (682)	3089-3770 (682)
trnK	CTT			3771-3841 (71)	3770-3840 (71)	3770-3840 (71)	3771-3841 (71)	3771-3841 (71)	3771-3841 (71)
trnD	GTC			3841-3907 (67)	3840-3906 (67)	3840-3906 (67)	3841-3907 (67)	3841-3907 (67)	3841-3907 (67)
ATP8		ATA	TAA	3908-4069 (162)	3907-4068 (162)	3907-4068 (162)	3908-4069 (162)	3908-4069 (162)	3908-4069 (162)
ATP6		ATG	TAA	4063-4740 (678)	4062-4739 (678)	4062-4739 (678)	4063-4740 (678)	4063-4740 (678)	4063-4740 (678)
COIII		ATT	TAA	4746-5543 (798)	4745-5542 (798)	4745-5542 (798)	4746-5543 (798)	4746-5543 (798)	4746-5543 (798)
trnG	TCC			5546-5611 (66)	5545-5610 (66)	5545-5610 (66)	5546-5611 (66)	5546-5611 (66)	5546-5611 (66)
ND3		ATT	TAA	5612-5965 (354)	5611-5964 (354)	5611-5964 (354)	5612-5965 (354)	5612-5965 (354)	5612-5965 (354)
trnA	TGC			6005-6072 (68)	6020-6087 (68)	6020-6087 (68)	6010-6077 (68)	6009-6076 (68)	6007-6074 (68)
trnR	TCG			6131-6194 (64)	6135-6198 (64)	6116-6179 (64)	6121-6184 (64)	6133-64196 (64)	6131-6194 (64)
trnN	GTT			6196-6262 (67)	6200-6266 (67)	6181-6247 (67)	6186-6252 (67)	6198-6264 (67)	6196-6262 (67)
trnS$_1$	GCT			6263-6329 (67)	6267-6333 (67)	6248-6314 (67)	6253-6319 (67)	6265-6331 (67)	6263-6329 (67)
trnE	TTC			6347-6411 (65)	6351-6415 (65)	6332-6396 (65)	6337-6402 (66)	6349-6413 (65)	6347-6411 (65)
trnF	GAA			6411-6477 (67)	6415-6481 (67)	6396-6462 (67)	6402-6468 (67)	6413-6479 (67)	6411-6477 (67)
ND5		ATT	TAA	6482-8221 (1740)	6486-8225 (1740)	6467-8206 (1740)	6473-8212 (1740)	6484-8223 (1740)	6482-8221 (1740)
trnH	GTG			8222-8288 (67)	8226-8292 (67)	8207-8273 (67)	8213-8279 (67)	8224-8290 (67)	8222-8288 (67)
ND4		ATT	TAA	8339-9685 (1347)	8347-9693 (1347)	8334-9680 (1347)	8340-9686 (1347)	8341-9687 (1347)	8339-9685 (1347)
ND4L		ATG	TAA	9679-9969 (291)	9687-9977 (291)	9674-9964 (291)	9680-9970 (291)	9681-9971 (291)	9679-9969 (291)
trnT	TGT			9974-10038 (65)	9982-10046 (65)	9969-10033 (65)	9975-10039 (65)	9976-10040 (65)	9974-10038 (66)
trnP	TGG			10039-10104 (66)	10047-10112 (66)	10034-10098 (65)	10040-10105 (66)	10041-10106 (66)	10039-10104 (66)
ND6		ATT	TAA	10107-10637 (531)	10115-10645 (531)	10101-10631 (531)	10108-10638 (531)	10109-10639 (531)	10107-10637 (531)

CytB		ATG[1]/ATC[2]	TAA	10680-11837 (1158)[1]	10690-11847 (1158)[1]	10671-11828 (1158)[1]	10683-11840 (1158)[1]	10682-11839 (1158)[1]	10680-11837 (1158)[1]
trnS$_2$	TGA			11840-11907 (68)	11850-11917 (68)	11831-11898 (68)	11843-11910 (68)	11842-11909 (68)	11840-11907 (68)
ND1		ATT	TAA	11932-12870 (939)	11942-12880 (939)	11923-12861 (939)	11935-12873 (939)	11934-12872 (939)	11932-12870 (939)
trnL$_1$	TAG			12871-12941 (71)	12881-12951 (71)	12862-12932 (71)	12874-12944 (71)	12873-12943 (71)	12871-12941 (71)
lrRNA				12942-14318 (1377)	12952-14329 (1378)	12933-14310 (1378)	12945-14322 (1378)	12944-14320 (1377)	12942-14318 (1377)
trnV	TAC			14319-14386 (68)	14330-14397 (68)	14311-14378 (68)	14323-14390 (68)	14321-14388 (68)	14319-14386 (68)
srRNA				14389-15169 (781)	14400-15180 (781)	14381-15161 (781)	14393-15173 (781)	14391-15171 (781)	14389-15169 (781)
A+T–rich region				15170-15663 (494)	15182-15676 (496)	15162-15664 (503)	15174-15667 (494)	15172-15665 (494)	15170-15664 (495)

Gene	Anticodon	Start codon	Stop codon	Jam319	Jam320
trnM	CAT			1-68 (68)	1-68 (68)
trnI	GAT			69-131 (63)	69-131 (63)
trnQ	TTG			130-198 (69)	130-198 (69)
ND2		ATA	TAA	246-1268 (1023)	246-1268 (1023)
trnW	TCA			1274-1343 (70)	1274-1343 (70)
trnC	GCA			1336-1402 (67)	1336-1402 (67)
trnY	GTA			1409-1474 (66)	1409-1474 (66)
COI		CGA	T-tRNA	1491-3021 (1531)	1491-3021 (1531)
trnL$_2$	TAA			3022-3088 (67)	3022-3088 (67)
COII		ATG	T-tRNA	3089-3770 (682)	3089-3770 (682)
trnK	CTT			3771-3841 (71)	3771-3841 (71)
trnD	GTC			3841-3907 (67)	3841-3907 (67)
ATP8		ATA	TAA	3908-4069 (162)	3908-4069 (162)
ATP6		ATG	TAA	4063-4740 (678)	4063-4740 (678)
COIII		ATT	TAA	4746-5543 (798)	4746-5543 (798)
trnG	TCC			5546-5611 (66)	5546-5611 (66)
ND3		ATT	TAA	5612-5965 (354)	5612-5965 (354)
trnA	TGC			6000-6067 (68)	6022-6089 (68)
trnR	TCG			6122-6185 (64)	6139-6202 (64)
trnN	GTT			6187-6253 (67)	6204-6270 (67)
trnS$_1$	GCT			6254-6320 (67)	6271-6337 (67)
trnE	TTC			6338-6402 (65)	6355-6419 (65)

trnF	GAA			6402-6468 (67)	6419-6485 (67)
ND5		ATT	TAA	6473-8212 (1740)	6490-8229 (1740)
trnH	GTG			8213-8279 (67)	8230-8296 (67)
ND4		ATT	TAA	8333-9679 (1347)	8348-9694 (1347)
ND4L		ATG	TAA	9673-9963 (291)	9688-9978 (291)
trnT	TGT			9968-10032 (65)	9983-10047 (65)
trnP	TGG			10033-10098 (66)	10048-10113 (66)
ND6		ATT	TAA	10101-10631 (531)	10116-10646 (531)
CytB		ATG[1]/ATC[2]	TAA	10673-11830 (1158)[1]	10688-11845 (1158)[1]
$trnS_2$	TGA			11833-11900 (68)	11848-11915 (68)
ND1		ATT	TAA	11925-12863 (939)	11940-12878 (939)
$trnL_1$	TAG			12864-12934 (71)	12879-12949 (71)
lrRNA				12935-14311 (1377)	12950-14326 (1377)
trnV	TAC			14312-14379 (68)	14327-14394 (68)
srRNA				14382-15162 (781)	14397-15177 (781)
A+T–rich region				15163-15657 (495)	15178-15666 (489)

Gene	Anticodon	Start codon	Stop codon	*Bombyx mandarina* (Gwangju)	*Bombyx mandarina* (Gapyeong)	*Bombyx mandarina* (Inje)	*Bombyx mandarina* (Sacheon)
trnM	CAT			1-68 (68)	1-68 (68)	1-68 (68)	1-68 (68)
trnI	GAT			67-132 (66)	67-132 (66)	67-132 (66)	67-132 (66)
trnQ	TTG			130-198 (69)	130-198 (69)	130-198 (69)	130-198 (69)
ND2		ATA	TAA	246-1268 (1023)	246-1268 (1023)	246-1268 (1023)	246-1268 (1023)
trnW	TCA			1274-1343 (70)	1274-1343 (70)	1274-1343 (70)	1274-1343 (70)
trnC	GCA			1336-1402 (67)	1336-1402 (67)	1336-1402 (67)	1336-1402 (67)
trnY	GTA			1409-1474 (66)	1409-1474 (66)	1409-1474 (66)	1409-1474 (66)
COI		CGA	T-tRNA	1491-3021 (1531)	1491-3021 (1531)	1491-3021 (1531)	1492-3022 (1531)
$trnL_2$	TAA			3022-3088 (67)	3022-3088 (67)	3022-3088 (67)	3023-3089 (67)
COII		ATG	T-tRNA	3089-3770 (682)	3089-3770 (682)	3089-3770 (682)	3090-3771 (682)
trnK	CTT			3771-3841 (71)	3771-3841 (71)	3771-3841 (71)	3772-3842 (71)
trnD	GTC			3841-3907 (67)	3841-3907 (67)	3841-3907 (67)	3842-3908 (67)
ATP8		ATA	TAA	3908-4069 (162)	3908-4069 (162)	3908-4069 (162)	3909-4070 (162)
ATP6		ATG	TAA	4063-4740 (678)	4063-4740 (678)	4063-4740 (678)	4064-4741 (678)
COIII		ATT	TAA	4747-5544 (798)	4746-5543 (798)	4746-5543 (798)	4747-5544 (798)

trnG	TCC			5547-5608 (62)	5546-5611 (66)	5546-5611 (66)	5547-5612 (66)
ND3		ATT	TAA	5613-5966 (354)	5612-5965 (354)	5612-5965 (354)	5613-5966 (354)
trnA	TGC			6020-6087 (68)	6015-6082 (68)	5997-6064 (68)	6016-6083 (68)
trnR	TCG			6131-6194 (64)	6126-6189 (64)	6100-6163 (64)	6131-6194 (64)
trnN	GTT			6196-6262 (67)	6191-6257 (67)	6165-6231 (67)	6196-6262 (67)
trnS$_1$	GCT			6263-6329 (67)	6258-6324 (67)	6232-6298 (67)	6263-6329 (67)
trnE	TTC			6345-6409 (65)	6341-6405 (65)	6315-6380 (66)	6345-6409 (65)
trnF	GAA			6409-6475 (67)	6405-6471 (67)	6380-6446 (67)	6409-6475 (67)
ND5		ATT	TAA	6480-8219 (1740)	6476-8215 (1740)	6451-8190 (1740)	6480-8219 (1740)
trnH	GTG			8220-8286 (67)	8216-8282 (67)	8191-8257 (67)	8220-8286 (67)
ND4		ATT	TAA	8368-9714 (1347)	8338-9684 (1347)	8315-9661 (1347)	8364-9710 (1347)
ND4L		ATG	TAA	9708-9998 (291)	9678-9968 (291)	9655-9945 (291)	9704-9994 (291)
trnT	TGT			10003-10067 (65)	9973-10037 (65)	9950-10014 (65)	9999-10063 (65)
trnP	TGG			10068-10133 (66)	10038-10103 (66)	10015-10080 (66)	10064-10129 (66)
ND6		ATT	TAA	10136-10666 (531)	10106-10636 (531)	10083-10613 (531)	10132-10662 (531)
CytB		ATG[1]/ATC[2]	TAA	10711-11868 (1158)[1]	10687-11844 (1158)[1]	10677-11834 (1158)[1]	10722-11880 (1158)[1]
trnS$_2$	TGA			11871-11938 (66)	11847-11914 (68)	11837-11904 (68)	11882-11949 (68)
ND1		ATT	TAA	11963-12901 (939)	11939-12877 (939)	11929-12867 (939)	11974-12912 (939)
trnL$_1$	TAG			12902-12972 (71)	12878-12948 (71)	12868-12938 (71)	12913-12983 (71)
lrRNA				12973-14350 (1378)	12949-14326 (1378)	12939-14315 (1377)	12984-14362 (1379)
trnV	TAC			14351-14425 (75)	14327-14401 (75)	14316-14390 (75)	14363-14437 (75)
srRNA				14428-15208 (781)	14404-15183 (780)	14393-15172 (780)	14440-15219 (780)
A+T–rich region				15209-15701 (493)	15184-15673 (490)	15173-15657 (485)	15220-15710 (491)

-, Not available.

Superscripts indicate identical start and stop codons. Values in parentheses indicate gene size (bp).

Gene names that are not underlined indicate a forward transcriptional direction, whereas underlining indicate a reverse transcriptional direction.
